# Supplementary material for: Engineered NIR-II fluorophores with ultralong-distance molecular packing for high-contrast deep lesion identification
Source: Nat Commun. 2023 Aug 18;14:5017. doi: 10.1038/s41467-023-40728-6 (PMC10439134; doi:10.1038/s41467-023-40728-6)
Supplement: Supplementary file 1 — Supplementary Information [file 41467_2023_40728_MOESM1_ESM.pdf]

Supplementary Information

Engineered NIR-II fluorophores with ultralong-distance molecular packing for high-contrast deep lesion identification

Zhe Feng[1,#], Yuanyuan Li[2,#], Siyi Chen[1], Jin Li[1], Tianxiang Wu[1], Yanyun Ying[3], Junyan Zheng[3], Yuhuang Zhang[1], Jianquan Zhang[4], Xiaoxiao Fan[1], Xiaoming Yu[3], Dan Zhang[3], Ben Zhong Tang[4]*, Jun Qian[1,*]

[1]State Key Laboratory of Modern Optical Instrumentations, Centre for Optical and Electromagnetic Research, College of Optical Science and Engineering, International Research Center for Advanced Photonics, Zhejiang University, Hangzhou 310058, China

[2]College of Veterinary Medicine, Jilin University, Changchun 130062, China

[3]Key Laboratory of Reproductive Genetics (Ministry of Education), Department of Reproductive Endocrinology, Women's Hospital, Zhejiang University School of Medicine, Hangzhou 310006, China

[4]Shenzhen Institute of Molecular Aggregate Science and Engineering, School of Science and Engineering, The Chinese University of Hong Kong, Shenzhen, 518172 China

#There authors contributed equally: Zhe Feng and Yuanyuan Li.

*e-mail: qianjun@zju.edu.cn; tangbenz@cuhk.edu.cn

Supplementary Methods

Materials. All the chemicals and reagents were purchased from chemical sources, and the solvents for chemical reactions were distilled before use. All air and moisture sensitive reactions were carried out in flame-dried glassware under a nitrogen atmosphere.

General measurements. The UV-Vis-NIR absorption spectra measurement were performed using a Shimadzu UV-3600 spectrophotometer. ^{1}H and ^{13}C spectra were recorded at room temperature on a Unity-400 NMR spectrometer using $CDCl_3$ as solvent and tetramethylsilane (TMS) as a reference. Mass spectra (MS) were measured with a GCT premier CAB048 mass spectrometer in MALDI-TOF mode. The photoluminescence (PL) spectra were measured via an Ideaoptics NIR2200 spectrofluorometer. It would be noteworthy that the PL spectra were measured with the 793 nm laser exciting on the edge of the cuvette, which minimized the absolute fluorescence loss caused by the water. The realistic intensity attenuation would be exacerbated in varying degrees in the different situations, due to the differentiated propagation and assorted compositions. Dynamic light scattering (DLS) was measured on a Zetasizer Nano-ZS analyzer. Transmission electron microscopy (TEM) images were acquired from a Tecnai™ Spirit transmission electron microscope with an accelerating voltage of 120 kV. Density functional theory calculations were carried out by the B3LYP/6G(d), Gaussian 09 package.

The imaging simulation *via* Monte Carlo method. The total thickness of tissue used in the simulation is infinite, with a length of 10 mm and a width of 10 mm. The refractive index of the tissue is set to 1.37, and scattering anisotropy is set to 0.9. In the simulation, the absorption coefficient of water is considered as the tissue absorption coefficient, and the reduced scattering coefficient is calculated by the following formula: $\mu_s' = 1.5\times10^3\,\lambda^{-1}$ (mm^{-1}). In the simulation, two lines with a length of 4 mm and a width of 180 μm are set as isotropic light sources and located orthogonally at a depth of 1 mm and 2 mm in the tissue, respectively. After a photon escapes from the tissue surface, it will travel through the imaging system and reach the detector. The imaging system consists of a single lens (with a focal length of 18 mm) and a detector. The system focuses on the line source on the more superficial site, with a magnification of 1. The detector is a 2D array plane with 512×512 resolution, and the pixel size is set to 20 μm. The number of photons used to generate each image is 10 million, and they are equally distributed over the two objects.

Analysis of the iFFT process. Since the random disturbance, which is non-negligible interference in the NIR photons detection, usually acts with high spatial frequency in one image, low-pass filtering was used for image denoising. Supplementary Fig. 28d shows the frequency domain map with the circular distribution of the 1400LP image, where the components with a specific spatial frequency are distributed in a circumference with a certain radius. With the decrease of the cut-off radius, high-frequency noise is constantly suppressed. However, detailed information of a picture also corresponds to high-spatial-frequency components which may be wasted through wave filtering without distinction. The diameters of three selected bright vessels in Supplementary Fig. 28a-c were measured after each inverse fast Fourier transform (iFFT).

Cytotoxicity assay. Ishikawa and CT26 were cultured in RPMI 1640 medium (C3010-0500, VivaCell, Shanghai XP Biomed Ltd., China) containing 10% fetal bovine serum (FBS) (Serana, Germany) and 1% penicillin/streptomycin. MB49 and 3T3 were grown in high-glucose DMEM medium (C3113-0500, VivaCell, Shanghai XP Biomed Ltd., China) supplemented with 10% FBS. All the cells were incubated in a humidified air with 5% CO_2 at 37 °C. Cells incubated in 96-well plates were treated with 0, 5, 10, 20, 40, 80 and 160 μM 2FT-*o*CB dots. Cell viability was assessed by a Cell Counting Kit-8 (BS350B, Biosharp, China) at 24 h post treatment following the manufacturer's instruction. The absorbance value was recorded at 450 nm.

***In vivo* biocompatibility test after intravenous injection.** The mice were divided into two groups including the control group (treated with 1×PBS, 200 μL) and the experimental group (treated with 2FT-*o*CB dots, 1 mg/mL, 200 μL). On the 1st and 28th day after the intravenous injection, the mice were anesthetized with isoflurane, and blood was collected from the orbital vein. 100 μL of whole blood was collected in the anticoagulation tubes for routine blood tests. The rest blood was collected in a centrifuge tube standing for 2 hours at room temperature, and then the serum was separated by centrifugation at 845 g. The blood routine was presented by a fully automated hematology analyzer (TEK8500 VET, TECOM). The blood biochemical assay was proved for creatinine (G034), total bilirubin (TBI01), and alanine aminotransferase (ALT01) measurement. The kits are from Ningbo Purebio Biotechnology CO., LTD., China. The heart, liver, spleen, lung, kidney, bladder, colon, and uterus were separated and weighed on the 28th day after the intravenous injection. The major and the application-related organs were then fixed in 10% formalin, processed routinely into paraffin, sectioned, stained with hematoxylin and eosin, and imaged for further analyses.

NIR-II QY test. The integrated emission intensities of the 2FT-*o*CB dots and IR-26 were recorded under identical excitation conditions. The intensities were plotted as a function of optical density (OD), respectively, and the slopes were calculated by linear fittings. The QY beyond 900 nm of the sample was calculated as follows:

$$QY=QY_{ref}\cdot\frac{Slope}{Slope_{ref}}\cdot\frac{n^2}{n_{ref}^2} \quad (1)$$

Where *QY* is the quantum yield of sample or reference, *n* is the index of refraction of the solvent, and *Slope* is the slope value in Fig. 2e. Subscript *ref* identifies the reference.

Then, the QY beyond 1400 nm was calculated as follows:

$$QY_{>1400\ nm}=\frac{N_{emit>1400\ nm}}{N_{abs}}=\frac{N_{emit>900\ nm}}{N_{abs}}\times\frac{N_{emit>1400\ nm}}{N_{emit>900\ nm}}=QY_{>900\ nm}\times\frac{I_{>1400\ nm}}{I_{>900\ nm}} \quad (2)$$

Where $I_{>1400\ nm}$ *and* $I_{>900\ nm}$ are the integrated fluorescence intensities of 2FT-*o*CB dots beyond 1400 nm and 900 nm, respectively, and $\frac{I_{>1400\ nm}}{I_{>900\ nm}}$ of 2FT-*o*CB dots could be calculated from the PL spectrum shown in Supplementary Fig. 39b.

Supplementary Figures

Synthetic procedures and characterization data for the compounds.

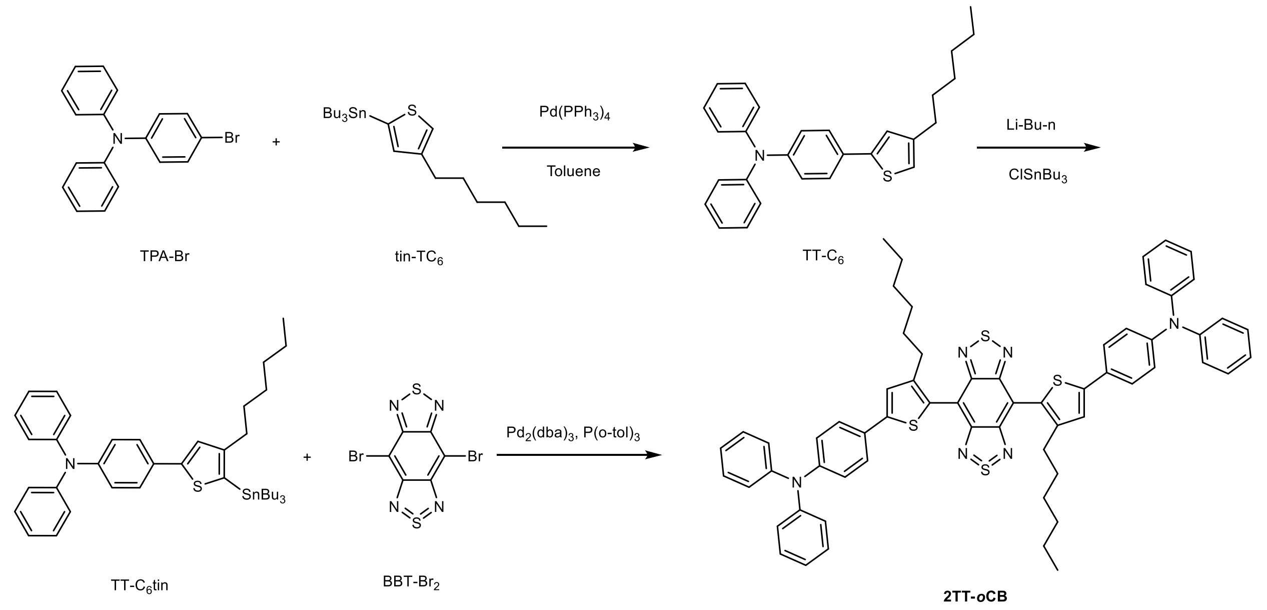

Supplementary Fig. 1. Synthetic route of **2TT-*o*CB**.

Synthetic route of TT-C_6.

Under N_2 atmosphere, TPA-Br (1 g, 3.1 mmol), tin-TC$_6$ (1.42 g, 3.1 mmol), Pd(PPh_3)$_4$ (180 mg, 0.15 mmol), and 20 mL toluene were added to a 100 mL predried two-necked flask. The mixture was refluxed for 24 h. After cooling down to room temperature, the solvent was removed by rotary evaporation. The crude product was purified by silica gel column to obtain the target molecule (yield, 70%). ^{1}H NMR (400 MHz, $CDCl_3$) δ 7.45 (2H, d, J = 8.8 Hz), 7.28-7.24 (4H, 8m), 7.12-7.10 (4H, m), 7.07-7.01 (5H, m), 2.60 (2H, t, J = 7.7 Hz), 1.67-1.60 (2H, m), 1.38- 1.27 (6H, m), 0.89 (3H, t, J = 6.7 Hz).

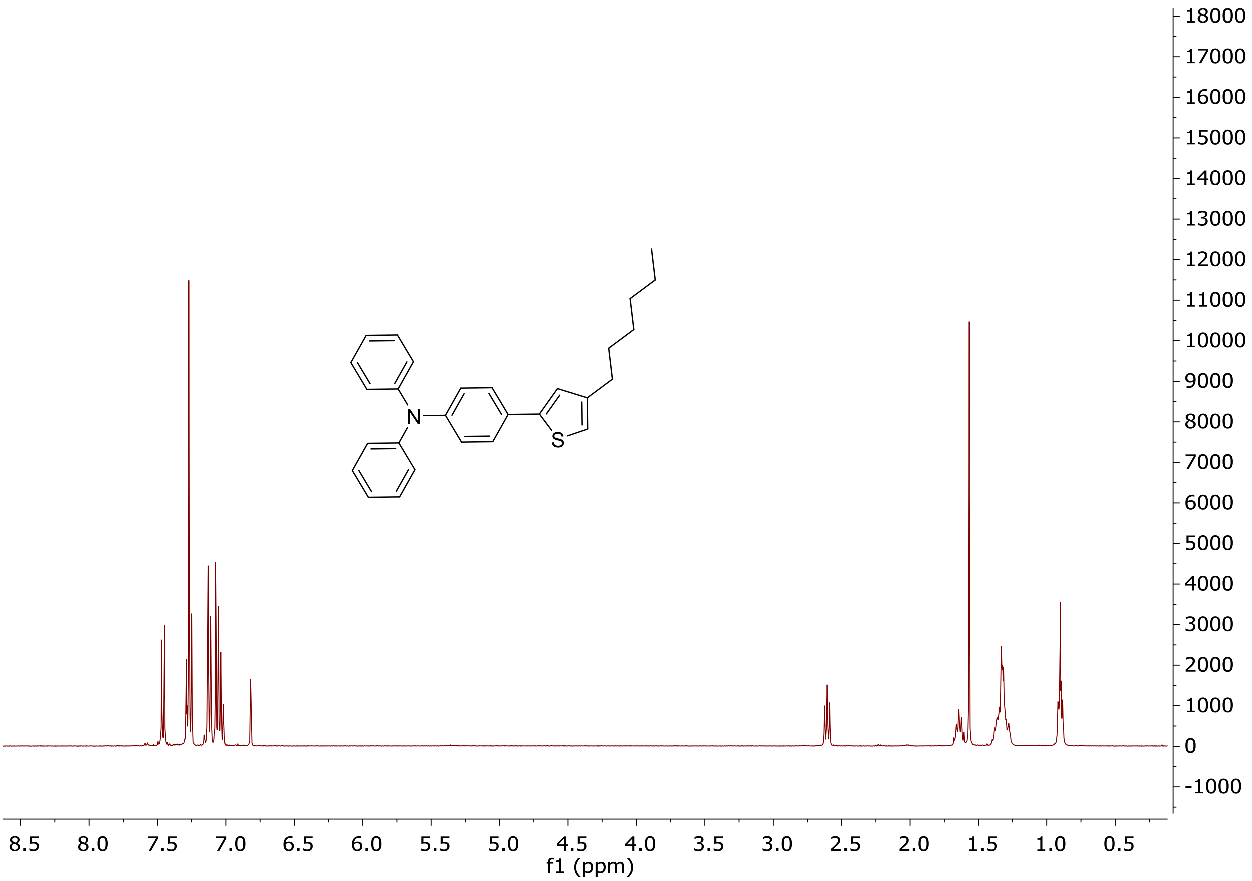

Supplementary Fig. 2. ^{1}H NMR spectrum of **TT-C$_6$**.

Synthetic route of TT-C$_6$tin.

nBuLi (2.3 mL, 5.8 mmol, 2.4 M in hexane) was added dropwise to a solution of TT-C$_6$ (2.16 g, 5.2 mmol) in THF (30 mL) at -78 °C. The reaction mixture was stirred 1 h at -78 °C. Then tributyltin chloride (1.8 g, 5.8 mmol) was added into the reaction at one portion. After stirring the mixture for 12 h at room temperature, KF solution was added to quench the reaction. The mixture was extracted with hexane three times, the combined organic phase was dried with Na_2SO_4. After removing the solvent, the product was used directly without further purification.

Synthetic route of 2TT-*o*CB.

A two-necked flask was charged with TT-C$_6$tin (0.7 g, 1 mmol), BBT-Br$_2$ (87 mg, 0.25 mmol), $Pd_2(dba)_3$ (22 mg, 0.025 mmol), P(*o*-tol)$_3$ (66 mg, 0.21 mmol), and degassed dry toluene (1.5 mL), and heated overnight under 110 °C. Upon cooling, the crude product was quenched with KF solution and extracted with DCM. The combined organic phase was dried with Na_2SO_4. After removing the solvent, the product was purified with silica column to obtain a dark green solid (yield: 45%). ^{1}H NMR (400 MHz, $CDCl_3$), δ (ppm) = 7.59-7.56 (4H, m), 7.37 (2H, s), 7.31-7.26 (8H, m), 7.16-7.12 (8H, m), 7.11-7.03 (8H, m), 2.61-2.57 (4H, t, J = 8Hz), 1.63, (4H, m), 1.15-1.10 (12H, m), 0.73 (6H, m). ^{13}C NMR (100 MHz, $CDCl_3$), δ (ppm): 152.6, 146.9, 146.8, 146.3, 145.0, 128.7, 127.5, 126.1, 124.1, 124.0, 122.7, 122.5, 115.4, 99.3, 30.8, 29.8, 29.6, 28.4, 21.8, 13.3. MS: m/z: [M]+ calcd for $C_{62}H_{56}N_6S_4$: 1012.3, found: 1012.3.

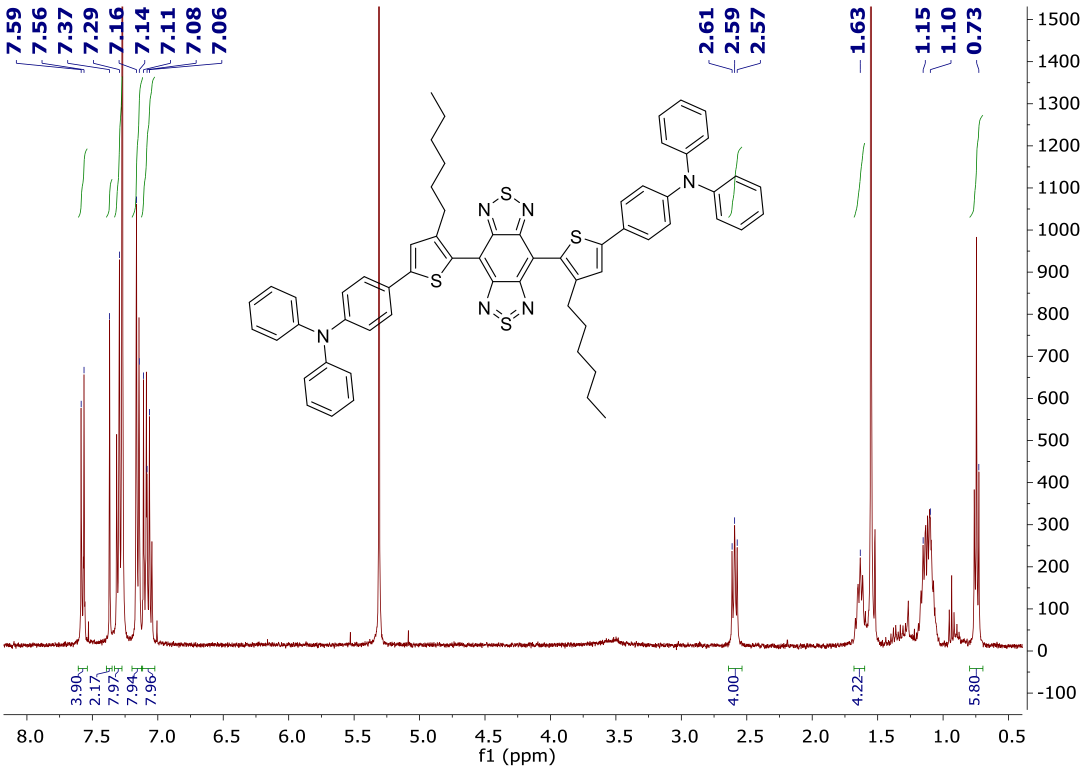

Supplementary Fig. 3. ^{1}H NMR spectrum of **2TT-*o*CB**.

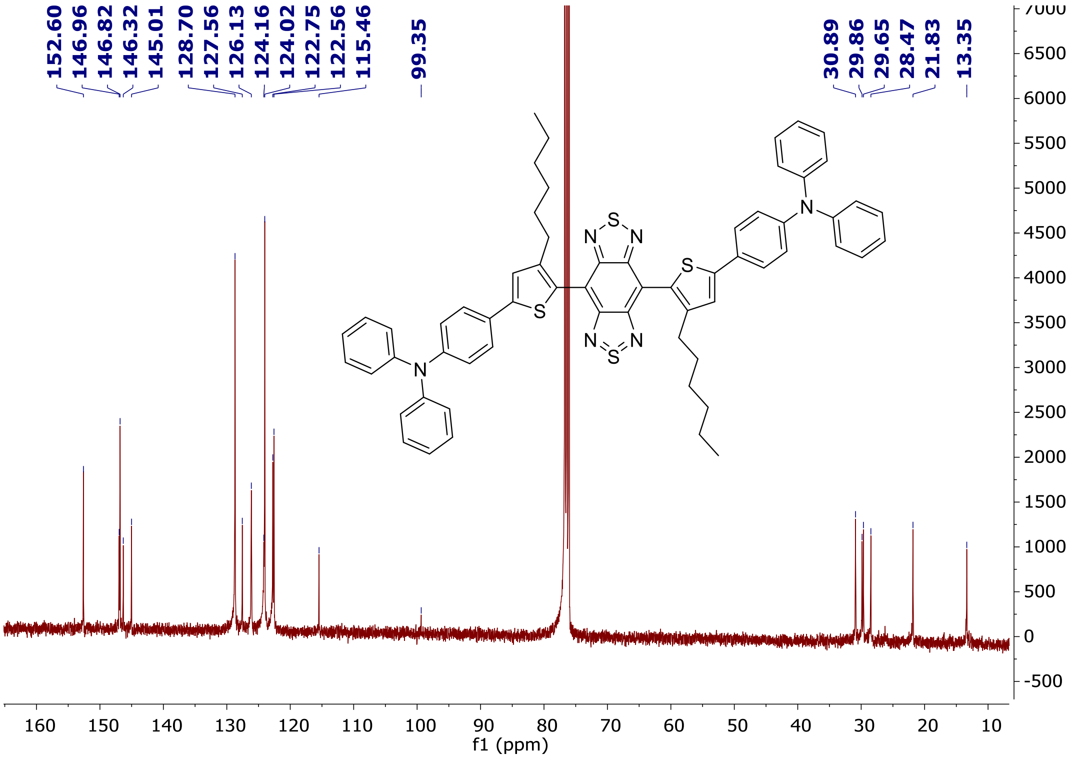

Supplementary Fig. 4. ^{13}C NMR spectrum of **2TT-*o*CB**.

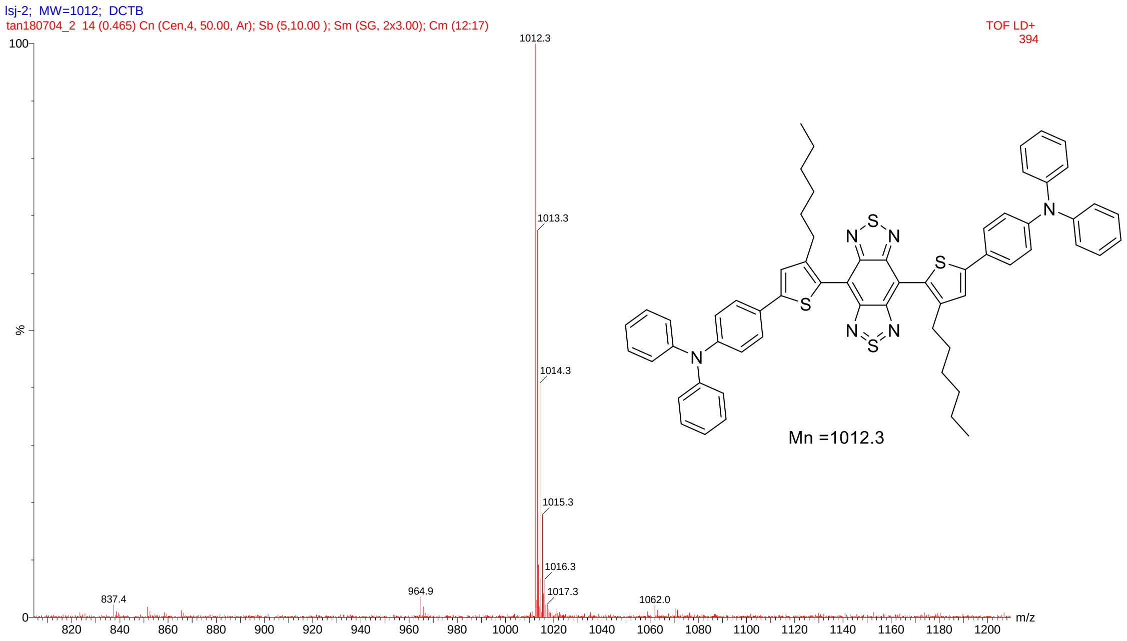

Supplementary Fig. 5. MALDI-TOF-MS spectrum of **2TT-*o*CB**.

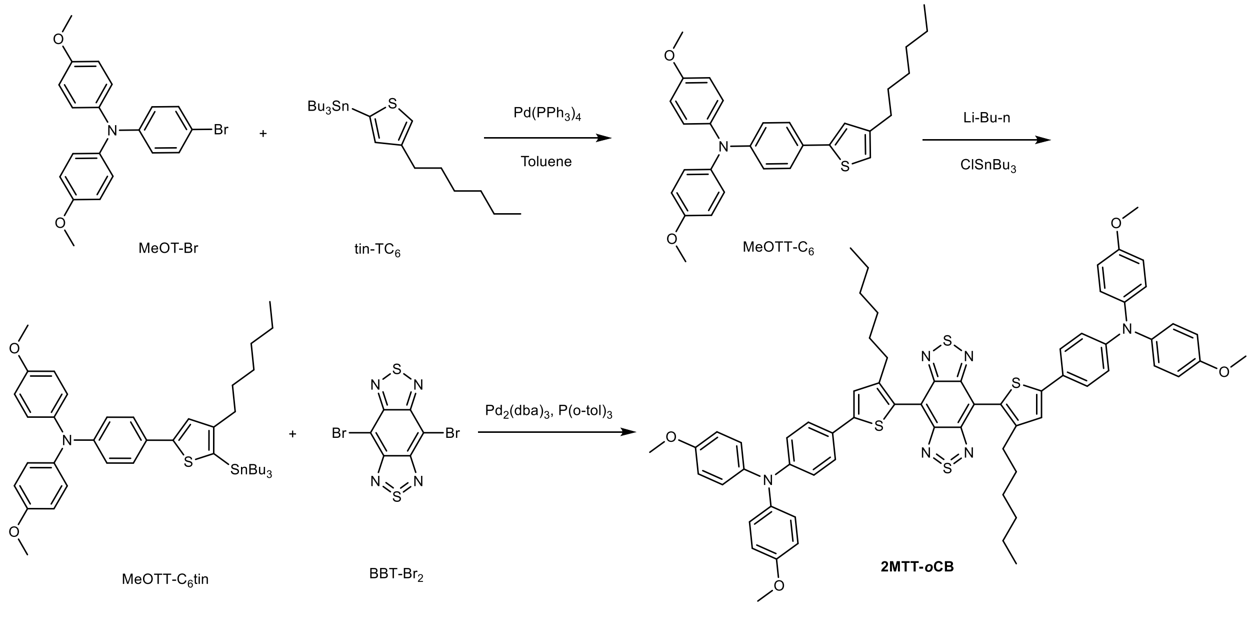

Supplementary Fig. 6. Synthetic route of **2MTT-*o*CB**.

Synthetic route of 2MTT-*o*CB.

The synthetic route to 2MTT-*o*CB was similar to that of 2TT-*o*CB by changing TPA-Br into MeOT-Br. ^{1}H NMR (400 MHz, $CDCl_3$), δ (ppm) = 7.54 (4H, m), 7.37 (2H, s), 7.12 (8H, m), 6.96 (12H, m), 6.88 (4H, m),

3.84 (12H, s), 2.62 (4H, m), 1.67 (4H, m), 1.12 (12H, m), 0.77 (6H, m). ^{13}C NMR (100 MHz, $CDCl_3$), δ (ppm): 156.05, 153.23, 148.53, 147.37, 145.62, 140.60, 127.76, 126.63, 126.27, 124.34, 120.23, 116.06, 114.77, 55.51, 31.54, 30.53, 30.30, 29.13, 22.49, 14.03.

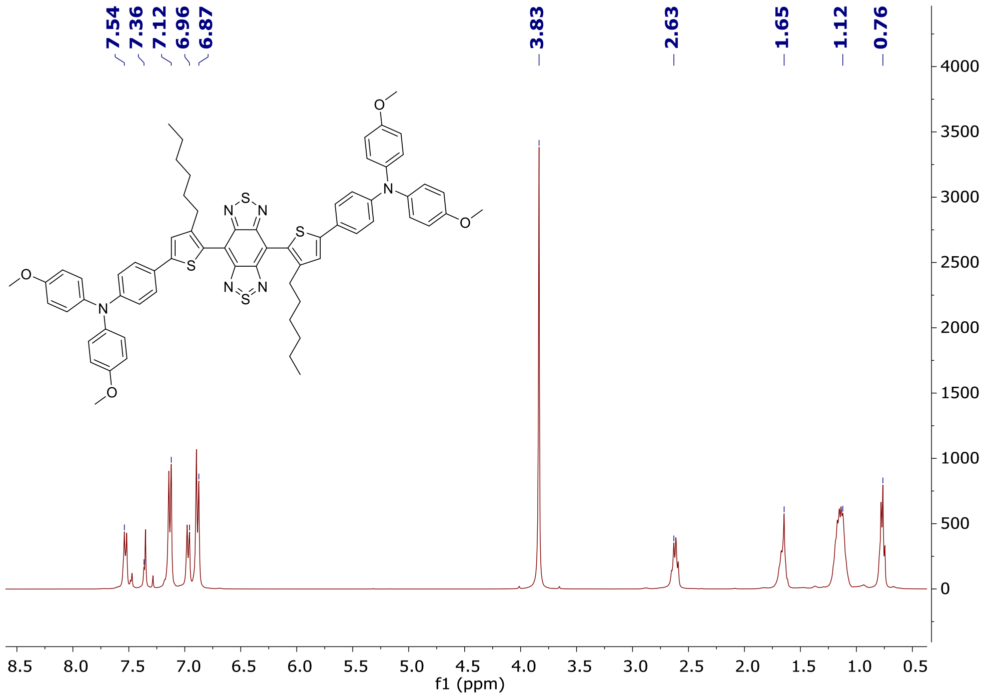

Supplementary Fig. 7. ^{1}H NMR spectrum of **2MTT-*o*CB**.

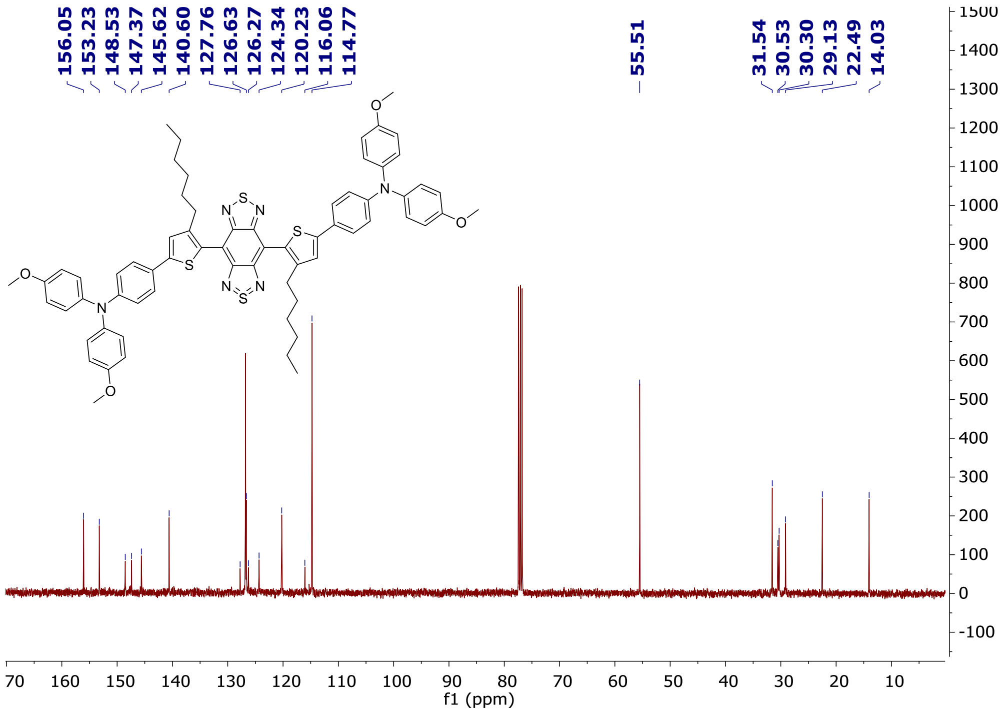

Supplementary Fig. 8. ^{13}C NMR spectrum of **2MTT-*o*CB**.

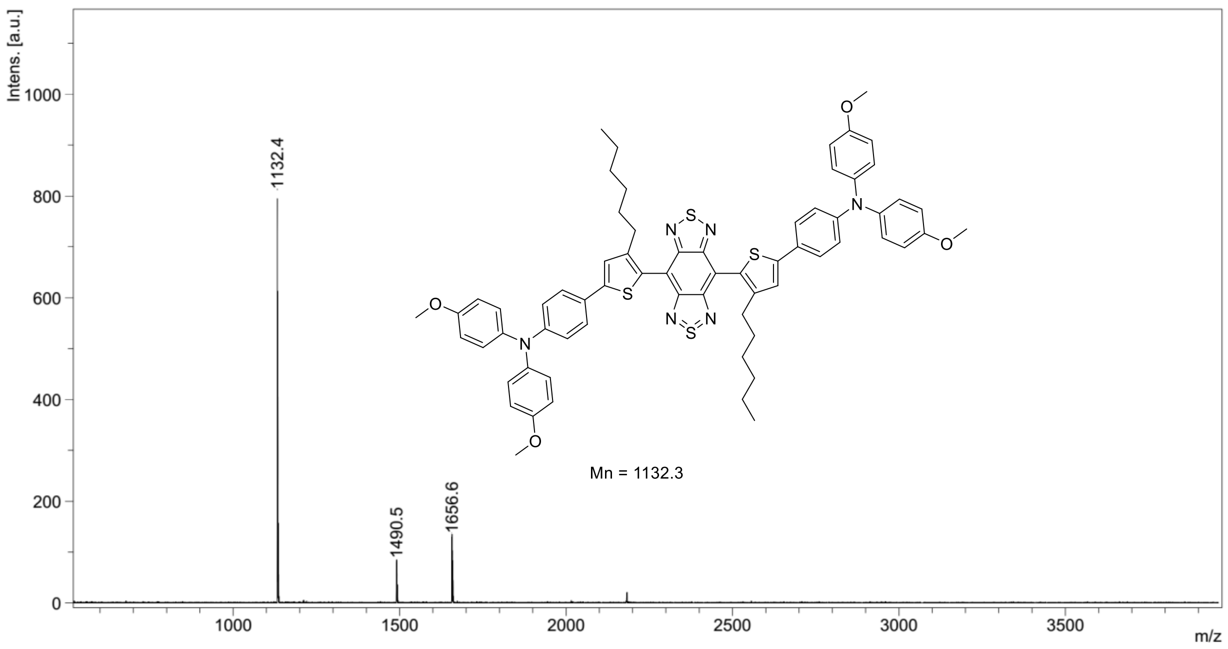

Supplementary Fig. 9. MALDI-TOF-MS spectrum of **2MTT-*o*CB**. a.u. here represents arbitrary units.

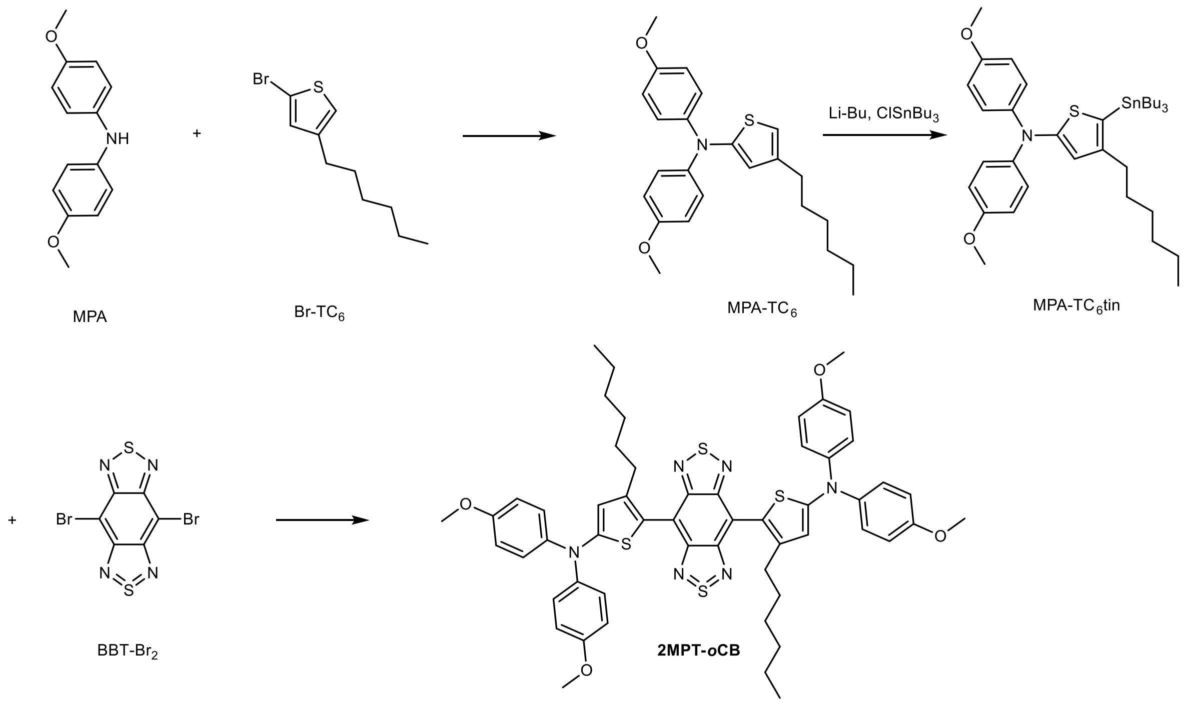

Supplementary Fig. 10. Synthetic route of **2MPT-*o*CB**.

Synthetic route of MPA-TC$_6$.

Bis(4-methoxyphenyl)amine MPA (0.23 g, 1 mmol), 2-bromo-4-hexylthiophene Br-TC$_6$ (0.25 g, 1 mmol), $Pd_2(dba)_3$ (46 mg, 0.05 mmol), P(t-Bu)$_3$ (0.8 mL, 0.4 mmol, 10w/v% in pentane), NaOBu-*t* (21.3 mg, 1.3 mmol) and toluene (10 mL) were added into a two-necked flask. The mixture was refluxed for 24 h under protection of nitrogen. After cooling down to room temperature, water was added to quench the reaction and the organic phase was extracted and dried. The crude product was purified by a silica gel column to obtain the product (yield: 70%). ^{1}H NMR (400 MHz, $CDCl_3$), δ (ppm) = 7.11 (4 H, m), 6.85 (4H, m), 6.45 (1H, s), 6.42 (1H, s), 3.83 (6H, s), 2.50 (2H, t, J = 8 Hz), 1.62 (2H, m), 1.36 (6H, m), 0.92 (3H, m).

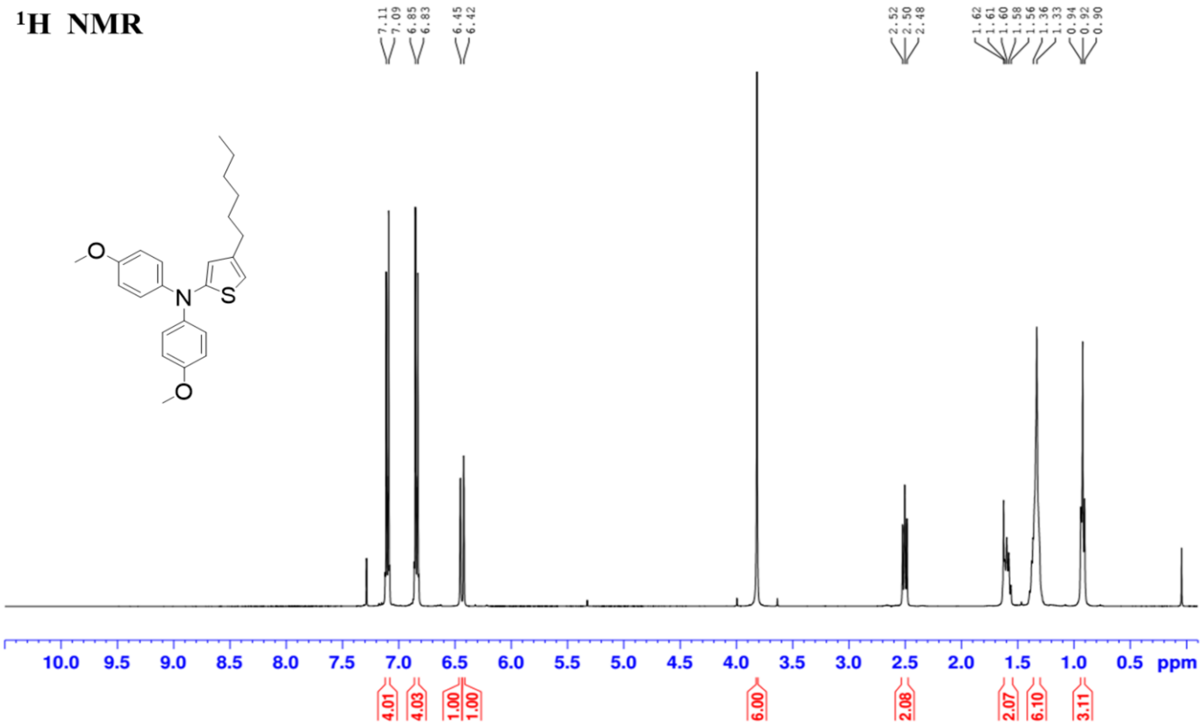

Supplementary Fig. 11. ^{1}H NMR spectrum of MPA-TC$_6$.

Synthetic route of MPA-TC$_6$tin.

nBuLi (2.3 mL, 5.8 mmol, 2.4 M in hexane) was added dropwise to a solution of MPA-TC$_6$ (2.05 g, 5.2 mmol) in THF (30 mL) at -78 °C. The reaction mixture was stirred 1 h at -78 °C. Then tributyltin chloride (1.8 g, 5.8 mmol) was added into the reaction at one portion. After stirring the mixture for 12 h at room temperature, KF solution was added to quench the reaction. The mixture was extracted with hexane three times, the combined organic phase was dried with Na_2SO_4. After removing the solvent, the product was used directly without further purification.

Synthetic route of 2MPT-*o*CB.

The synthetic route to 2MPT-*o*CB was similar to that of 2MTT-*o*CB by changing MeOTT-C$_6$tin into MPA-TC$_6$tin. ^{1}H NMR (400 MHz, $CDCl_3$), δ (ppm) = 7.32 (8H, d, J = 8Hz), 6.90 (8H, d, J = 8Hz), 6.49 (2H, m), 3.82 (12H, s), 2.50 (4H, t, J = 8Hz), 1.52 (4H, m), 1.12 (12H, m), 0.76 (6H, m). ^{13}C NMR (100 MHz,

$CDCl_3$), δ (ppm): 157.37, 156.56, 153.20, 144.29, 140.74, 126.14, 115.14, 114.72, 114.64, 55.50, 31.54, 31.05, 29.21, 22.52, 14.02. MS: m/z: [M]+ calcd for $C_{54}H_{56}N_6S_4O_4$: 980.3, found: 980.3.

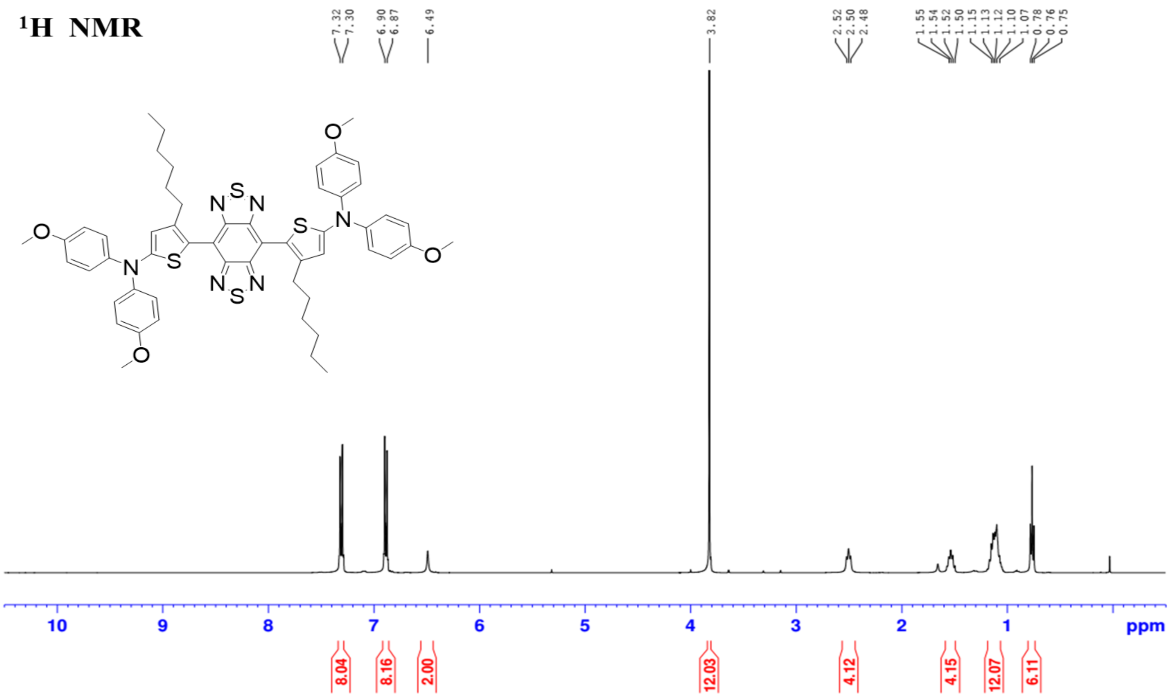

Supplementary Fig. 12. ¹H NMR spectrum of **2MPT-*o*CB**.

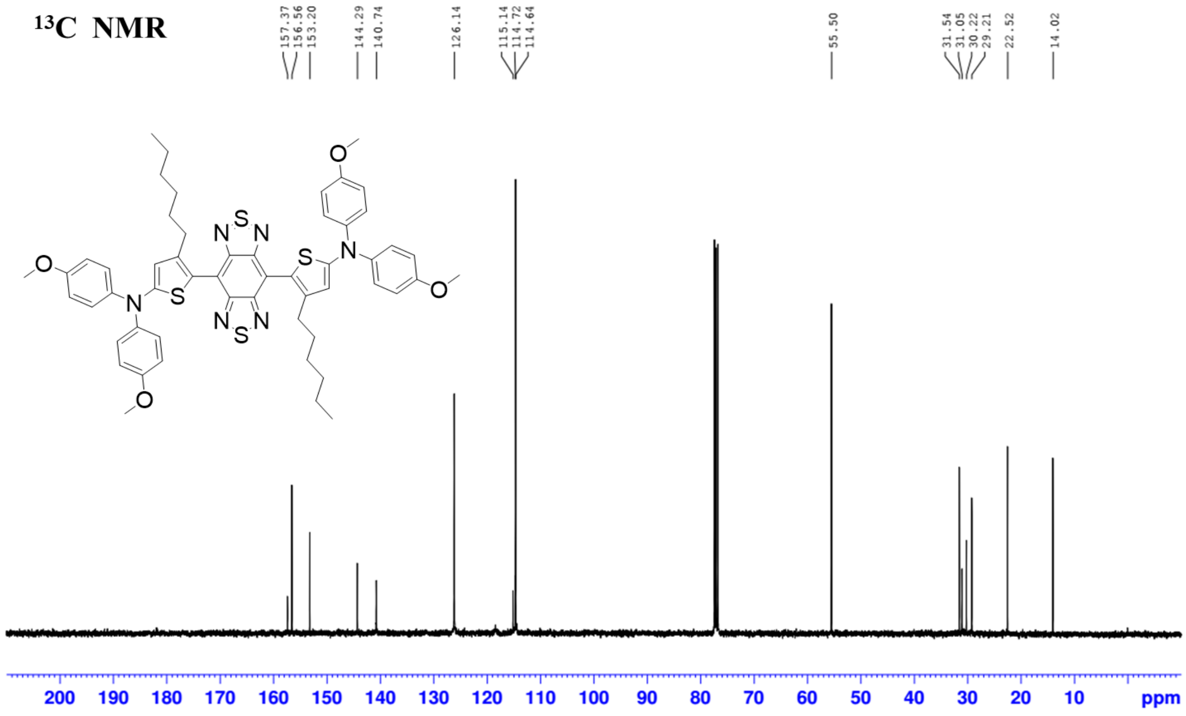

Supplementary Fig. 13. ¹³C NMR spectrum of **2MPT-*o*CB**.

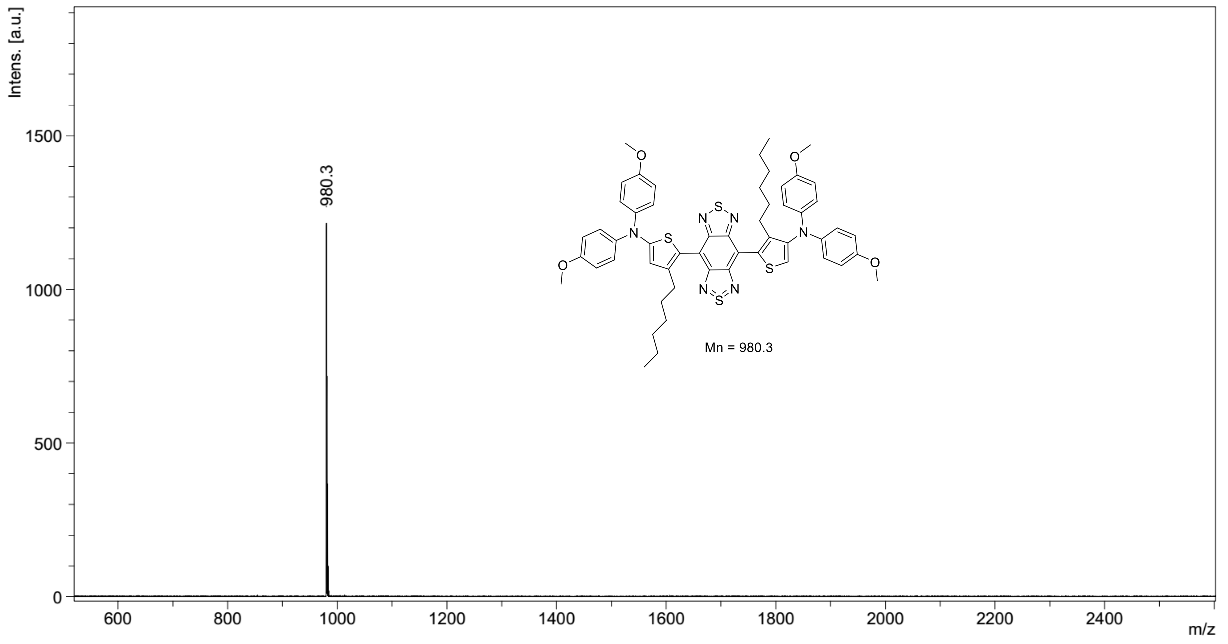

Supplementary Fig. 14. MALDI-TOF-MS spectrum of **2MPT-*o*CB**. a.u. here represents arbitrary units.

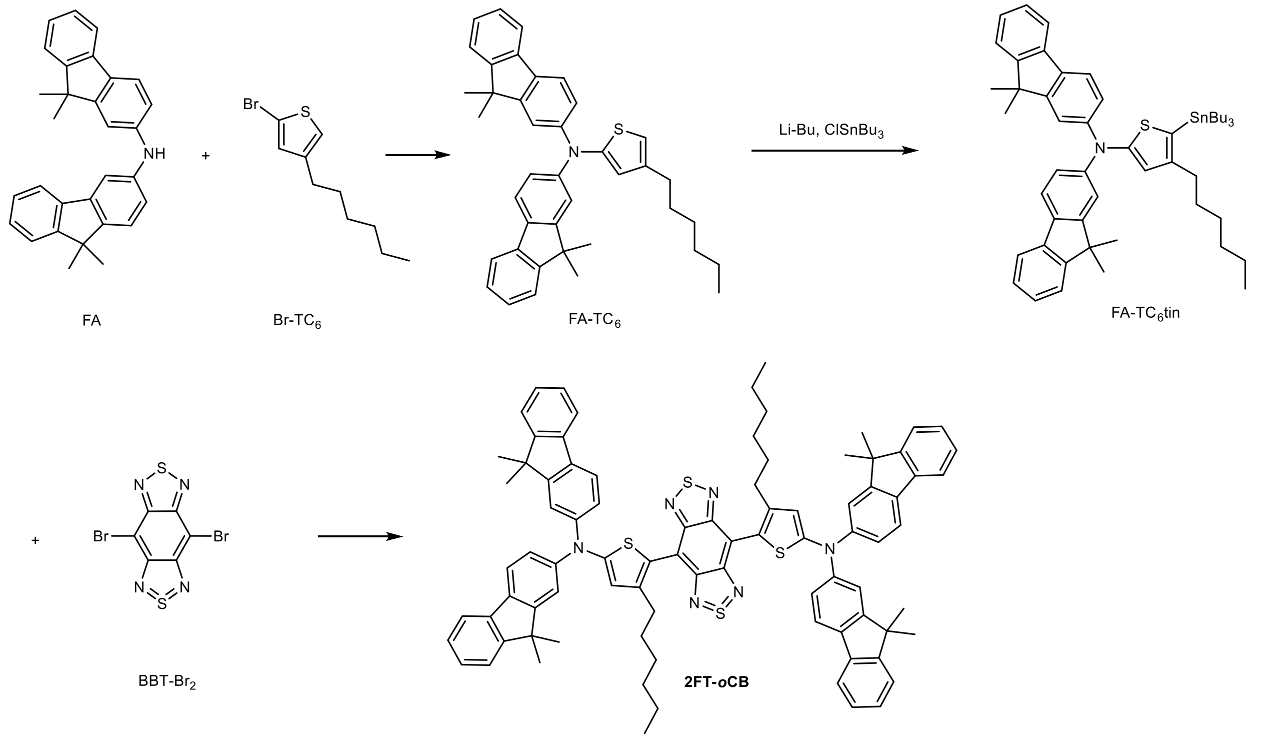

Supplementary Fig. 15. Synthetic route of **2FT-*o*CB**.

Synthetic route of FA-TC$_6$.

2-bromo-4-hexylthiophene Br-TC$_6$ (0.25 g, 1 mmol), FA (0.56 g, 1 mmol), $Pd_2(dba)_3$ (46 mg, 0.05 mmol), $P(t\text{-}Bu)_3$ (0.8 mL, 0.4 mmol, 10w/v% in pentane), NaOBu-*t* (21.3 mg, 1.3 mmol) and toluene (10 mL) were added into a two-necked flask. The mixture was refluxed for 24 h under protection of nitrogen. After cooling down to room temperature, water was added to quench the reaction and the organic phase was extracted and dried. The crude product was purified by a silica gel column to obtain the product (yield: 71%). ^{1}H NMR (400 MHz, $CDCl_3$), δ (ppm) = 7.68 (8H, m), 7.44 (2H, m), 7.32 (2H, m), 7.16 (2H, m), 6.68 (1H, s), 6.61 (1H, s), 2.58 (2H, t, J = 8Hz), 1.64 (2H, m), 1.46 (12H, s), 1.35 (6H, m), 0.93 (3H, m).

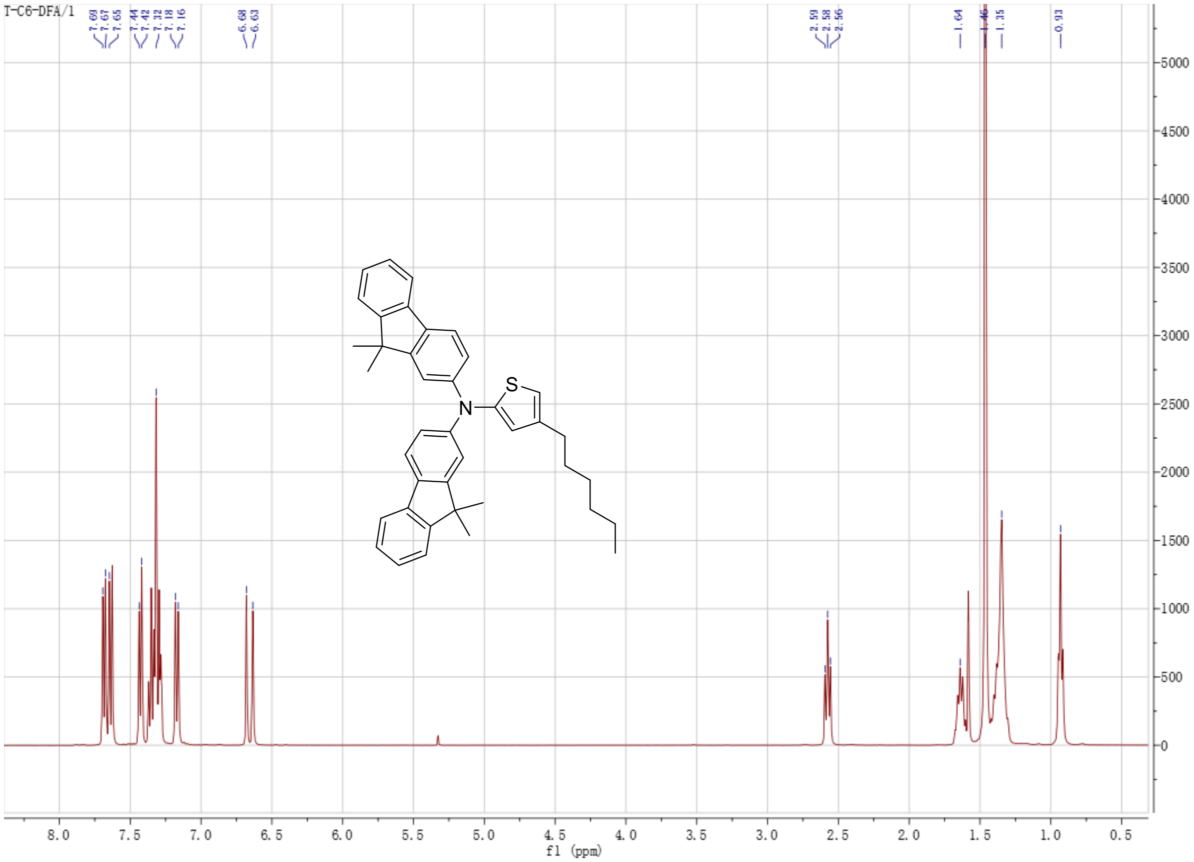

Supplementary Fig. 16. ^{1}H NMR spectrum of FA-TC$_6$.

Synthetic route of FA-TC$_6$tin.

nBuLi (2.3 mL, 5.8 mmol, 2.4 M in hexane) was added dropwise to a solution of FA-TC$_6$ (2.94 g, 5.2 mmol) in THF (30 mL) at -78 °C. The reaction mixture was stirred 1 h at -78 °C. Then tributyltin chloride (1.8 g, 5.8 mmol) was added into the reaction at one portion. After stirring the mixture for 12 h at room temperature, KF solution was added to quench the reaction. The mixture was extracted with hexane three times, the combined organic phase was dried with Na_2SO_4. After removing the solvent, the product was used directly without further purification.

Synthetic route of 2FT-*o*CB.

The synthetic route to **2FT-*o*CB** was similar to that of **2MPT-*o*CB** by changing MPA-TC$_6$tin into FA-TC$_6$tin. ^{1}H NMR (400 MHz, $CDCl_3$), δ (ppm) = 7.77 (4H, m), 7.54 (2H, s), 7.47-7.28 (22H, m), 2.63 (4H,

t, J = 8Hz), 1.63 (4H, m), 1.52 (24H, m), 1.21 (12H, m), 0.80 (6H, m). ^{13}C NMR (100 MHz, $CDCl_3$), δ (ppm): 155.60, 155.01, 153.69, 153.28, 146.99, 144.07, 138.84, 135.32, 127.09, 126.80, 123.18, 122.58, 120.71, 119.66, 118.60, 115.37, 68.02, 46.99, 31.60, 31.12, 30.40, 29.20, 27.11, 25.68, 22.54, 14.07. MS: m/z: [M]+ calcd for $C_{86}H_{80}N_6S_4$: 1324.5, found: 1324.5.

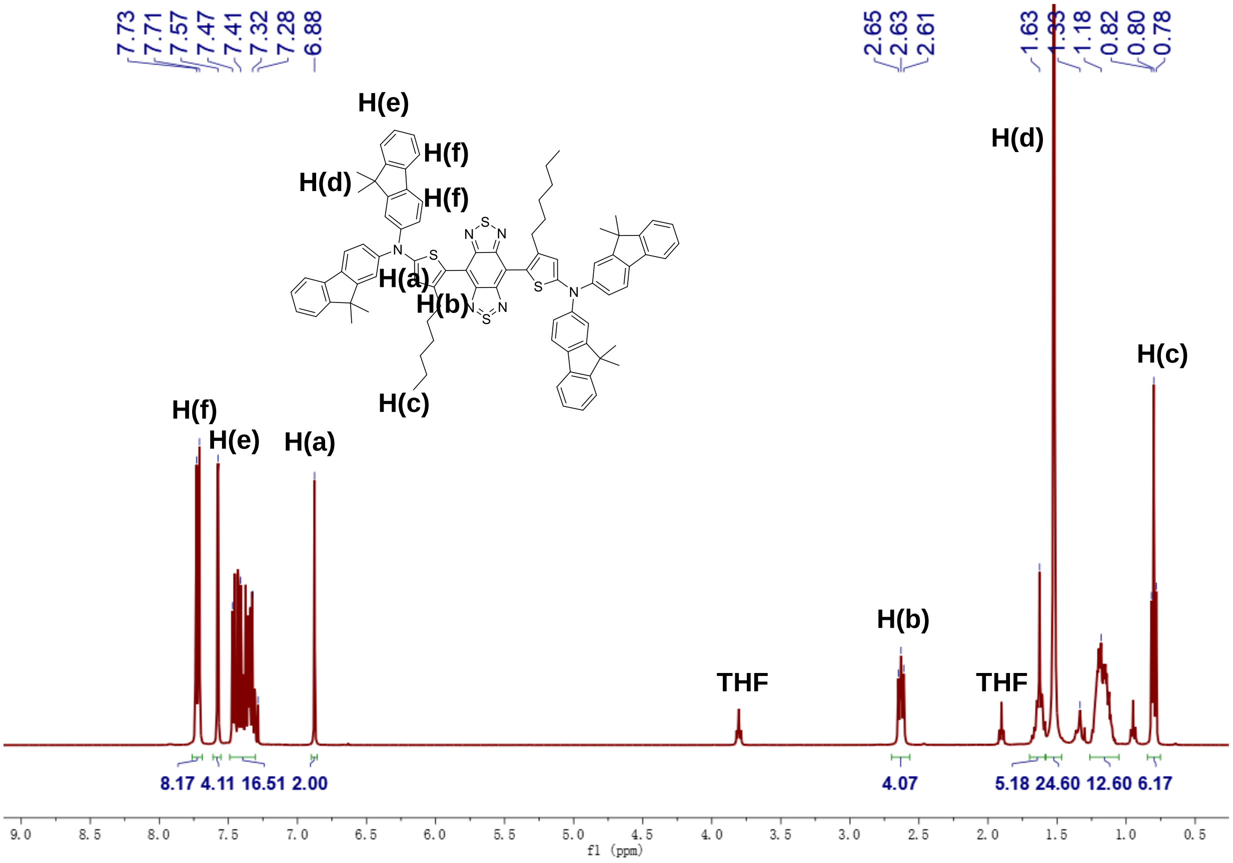

Supplementary Fig. 17. ^{1}H NMR spectrum of **2FT-*o*CB**.

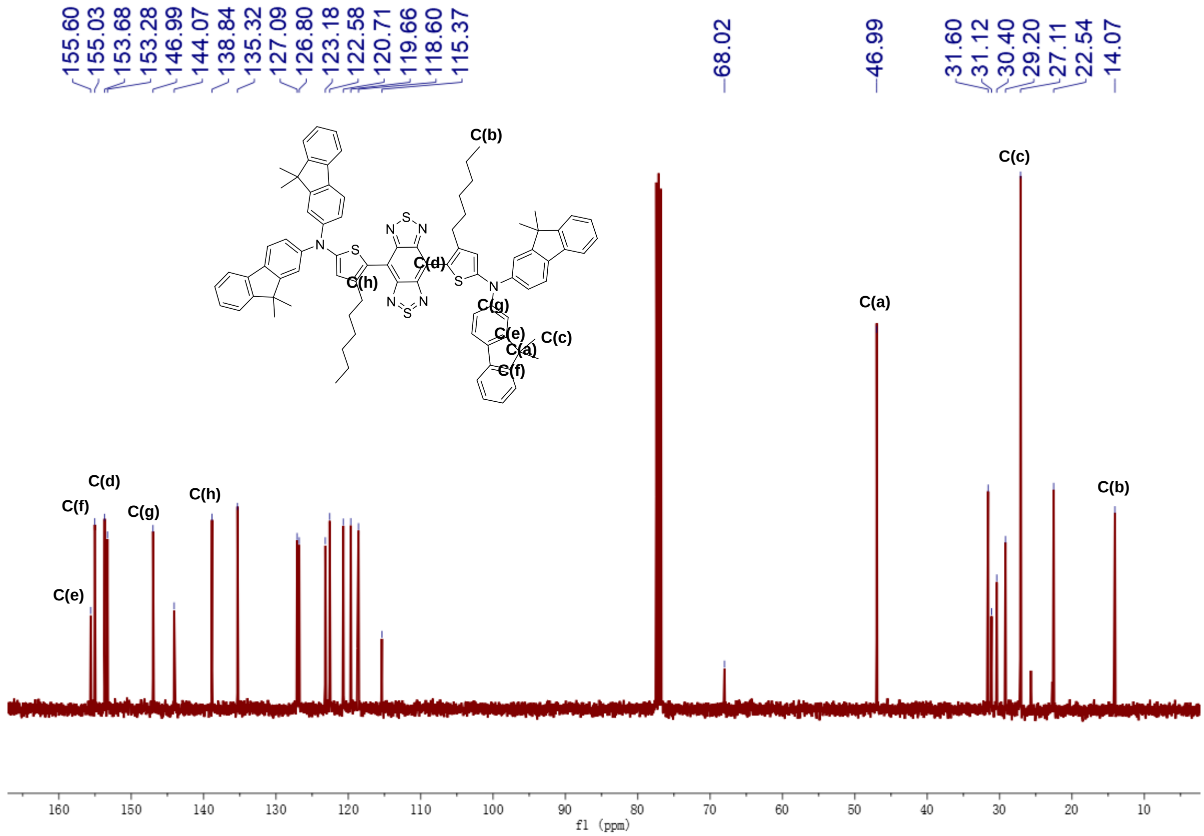

Supplementary Fig. 18. ^{13}C NMR spectrum of **2FT-*o*CB**.

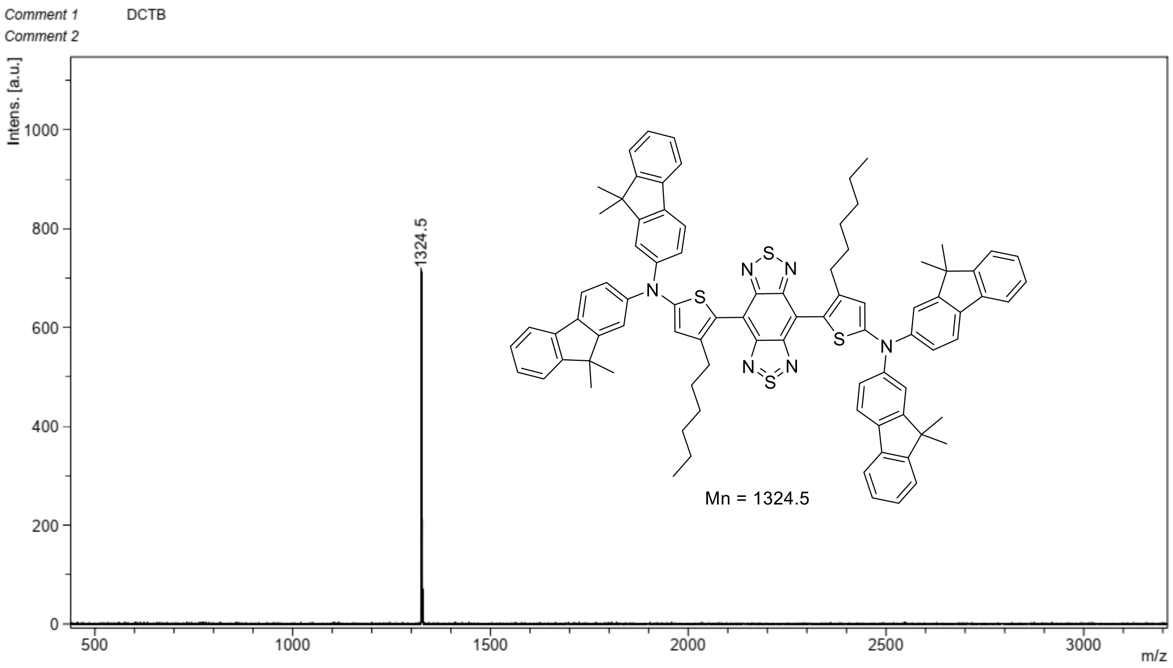

Supplementary Fig. 19. MALDI-TOF-MS spectrum of **2FT-*o*CB**. a.u. here represents arbitrary units.

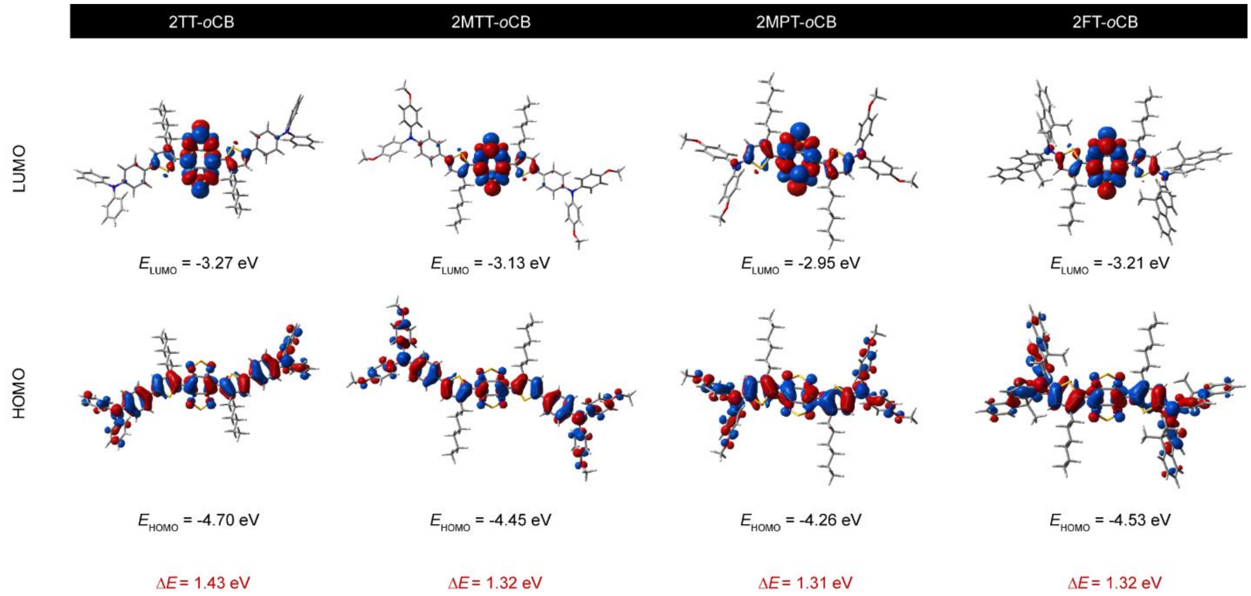

Supplementary Fig. 20. The frontier molecular orbitals of the LUMO and the HOMO determined at the B3LYP/6-311g(d,p) level of theory.

The HOMO of the molecules is delocalized along the whole molecule, revealing an excellent molecular conjugation. While the LUMO is primarily located on the BBTD core. The energy gap between HOMO and LUMO for 2TT-*o*CB (1.43 eV), 2MTT-*o*CB (1.32 eV), 2MPT-*o*CB (1.31 eV) and 2FT-*o*CB (1.32 eV) decreases with an increase of D-A interactions, indicating enhanced absorption intensity. Notably, these low energy gaps are beneficial for strong absorption in the NIR biological window.

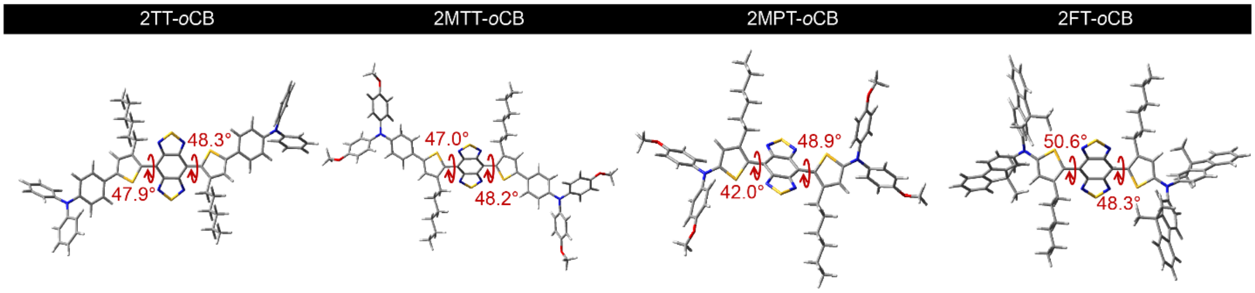

Supplementary Fig. 21. The optimized S_0 geometries of the four molecules (2TT-*o*CB, 2MTT-*o*CB, 2MPT-*o*CB and 2FT-*o*CB).

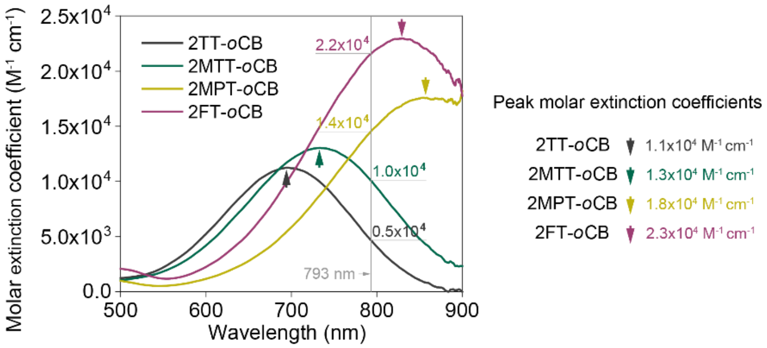

Supplementary Fig. 22. The spectra of the four AIEgens in THF showing the molar extinction coefficients at various wavelengths (especially the molar extinction coefficients at 793 nm and the peak molar extinction coefficients).

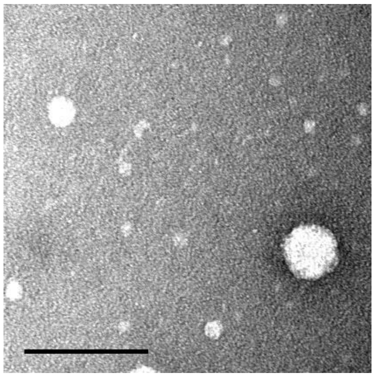

Supplementary Fig. 23. The representative TEM image of the 2FT-*o*CB dots negatively stained with uranyl acetate in three independent experiments. Scale bar, 100 nm.

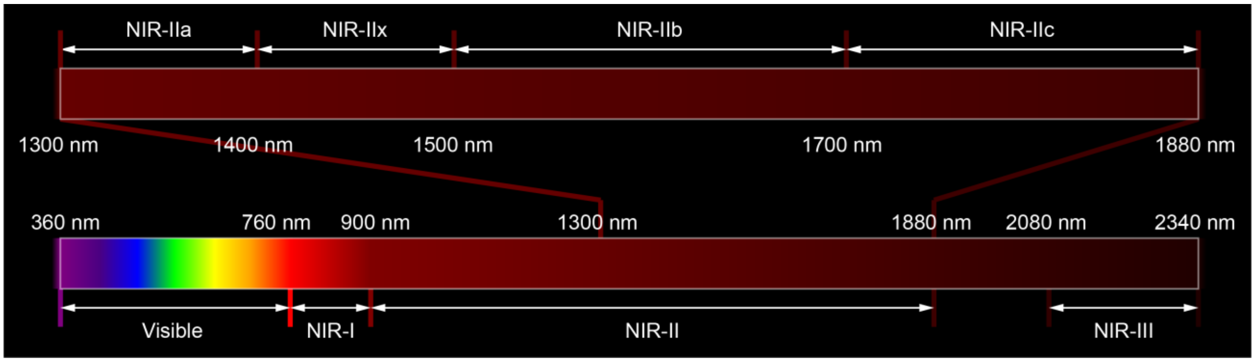

Supplementary Fig. 24. The visible-NIR imaging windows.

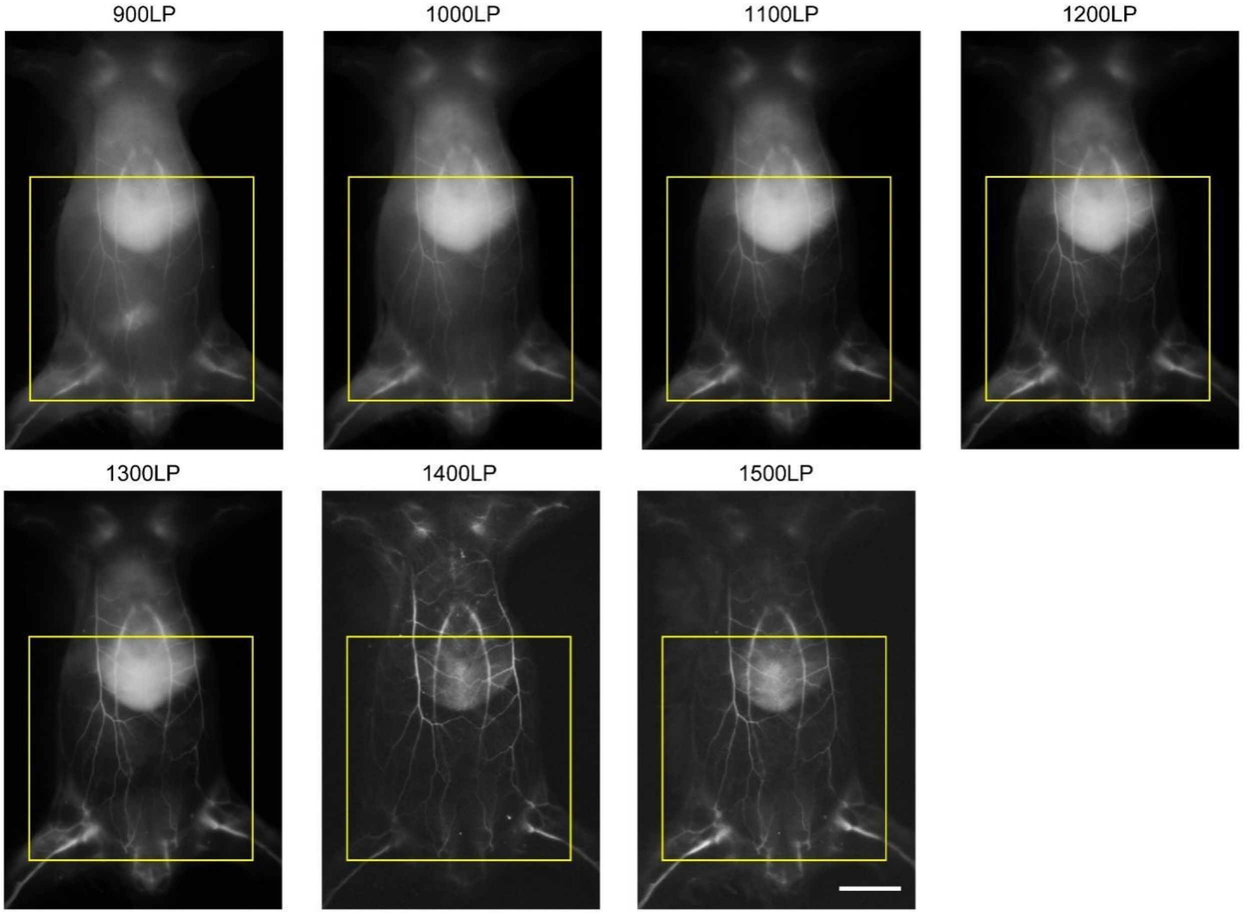

Supplementary Fig. 25. The NIR-II fluorescence whole-body vessel imaging in a mouse in different spectral regions. The selected areas with yellow squares were the original images of the FFT results in Supplementary Fig. 26. Scale bar, 10 mm.

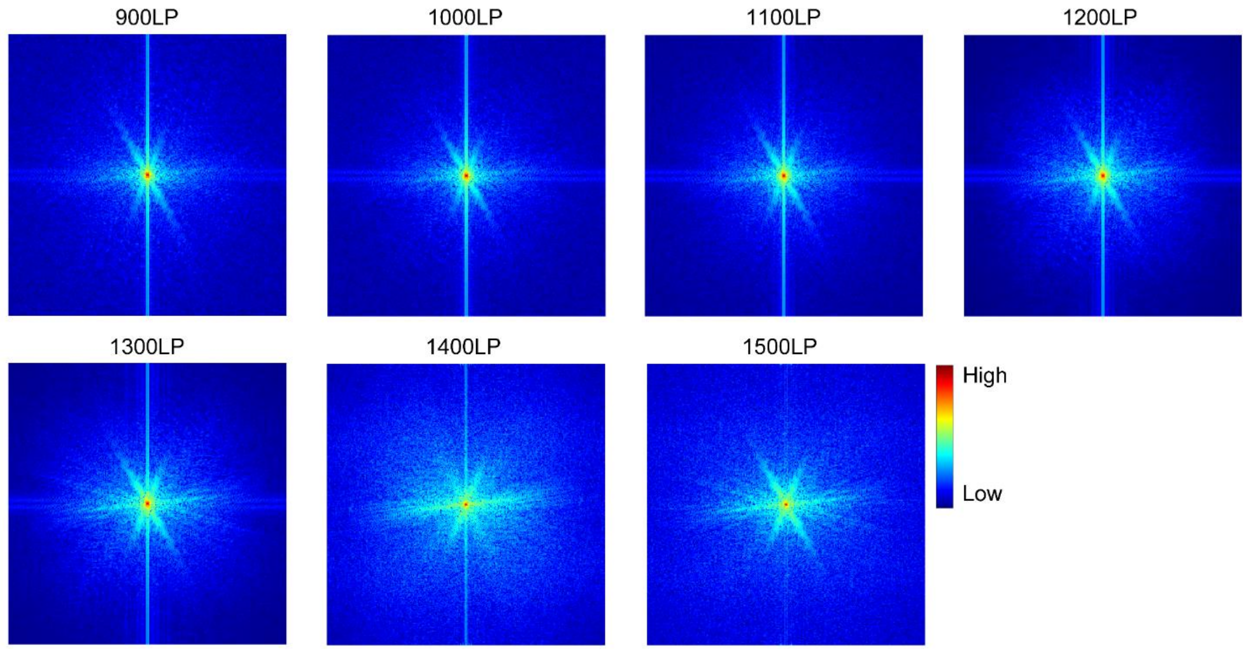

Supplementary Fig. 26. The FFT results of the NIR-II fluorescence vessel images in Supplementary Fig. 25. The spatial frequency gradually increases outward from the center of the map, and the color bar indicates the intensity.

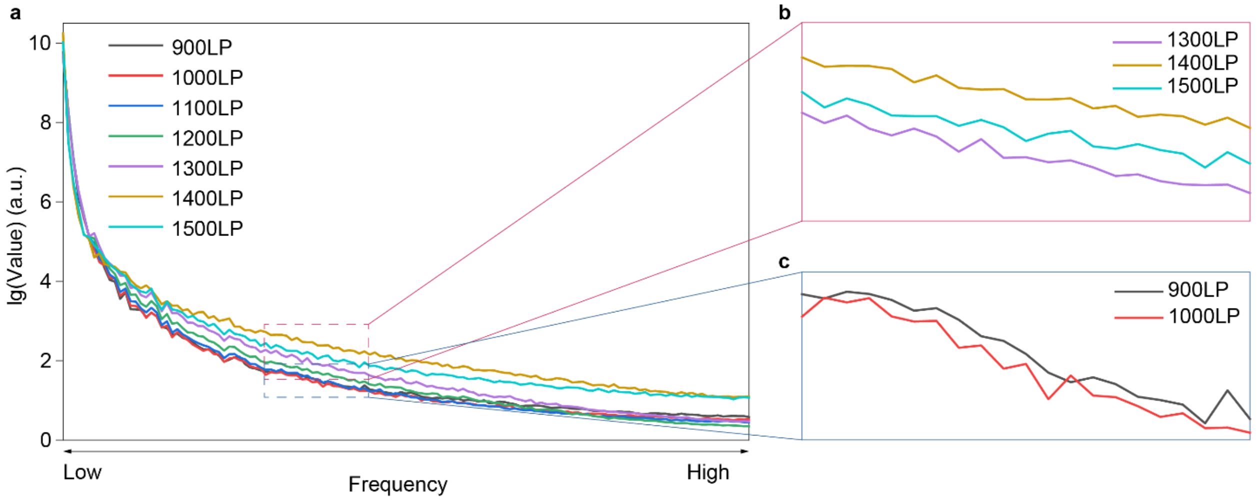

Supplementary Fig. 27. **The frequency distributions of the FFT results. (a)** The frequency distributions with 900LP, 1000LP, 1100LP, 1200LP, 1300LP, 1400LP, and 1500LP detection. **(b)** The enlarged region for direct comparison of 1300LP, 1400LP, and 1500LP imaging. **(c)** The enlarged region for direct comparison of 900LP and 1000LP imaging. a.u. here represents arbitrary units.

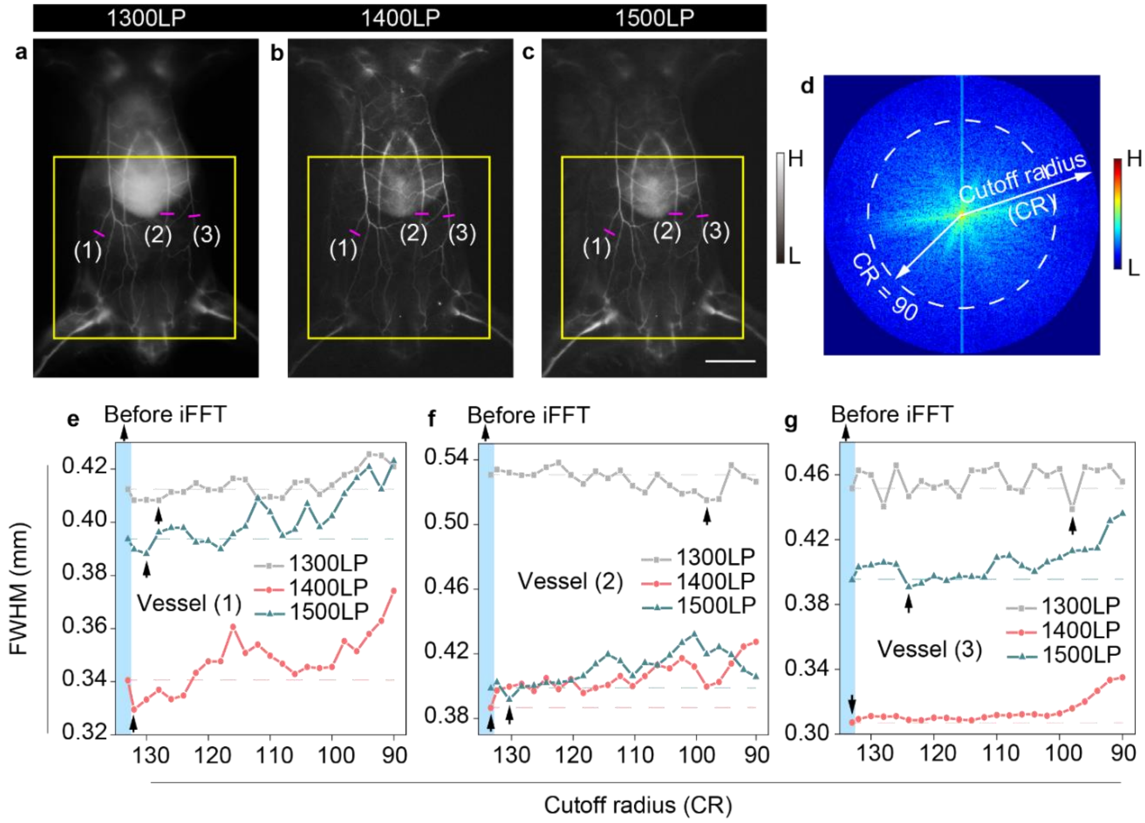

Supplementary Fig. 28. **The iFFT analysis.** The (a) 1300LP, (b) 1400LP and (c) 1500LP whole-body fluorescence images in the same mouse after intravenous injection of 2FT-*o*CB dots. Scale bar, 10 mm. (d) The FFT result of the 1400-nm LP image where the points in a circumference possessed the same spatial frequency and the cut-off radius represented the cut-off frequency of short-pass filtering. The FWHMs of the (e) vessel (1), (f) vessel (2), and (g) vessel (3) in (a-c) without any filtering (before iFFT) and after short-pass filtering with decreasing cut-off radius. The black arrows pointed to the minimal measured diameters of one vessel in the image.

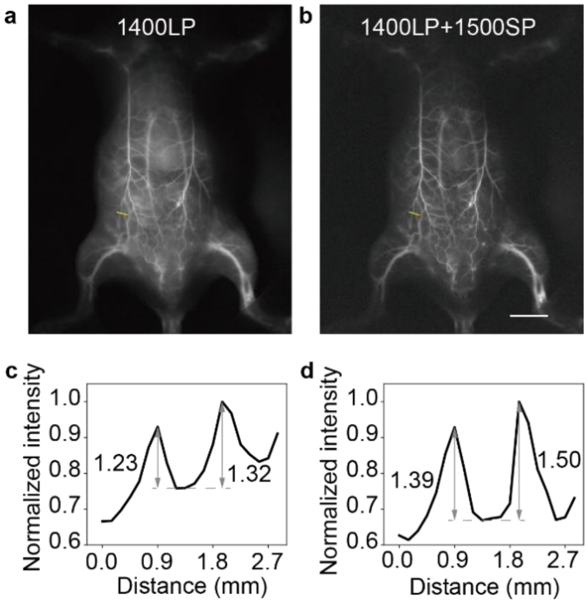

Supplementary Fig. 29. **The *in vivo* vessel imaging using water-based solution of the 2FT-*o*CB dots.** The images in (a) 1400-1700 nm window and (b) 1400-1500 nm window. Scale bar, 10 mm. (c) The cross-sectional fluorescence intensity profiles along the yellow line in (a). (d) The cross-sectional fluorescence intensity profiles along the yellow line in (b). The numbers show the SBRs.

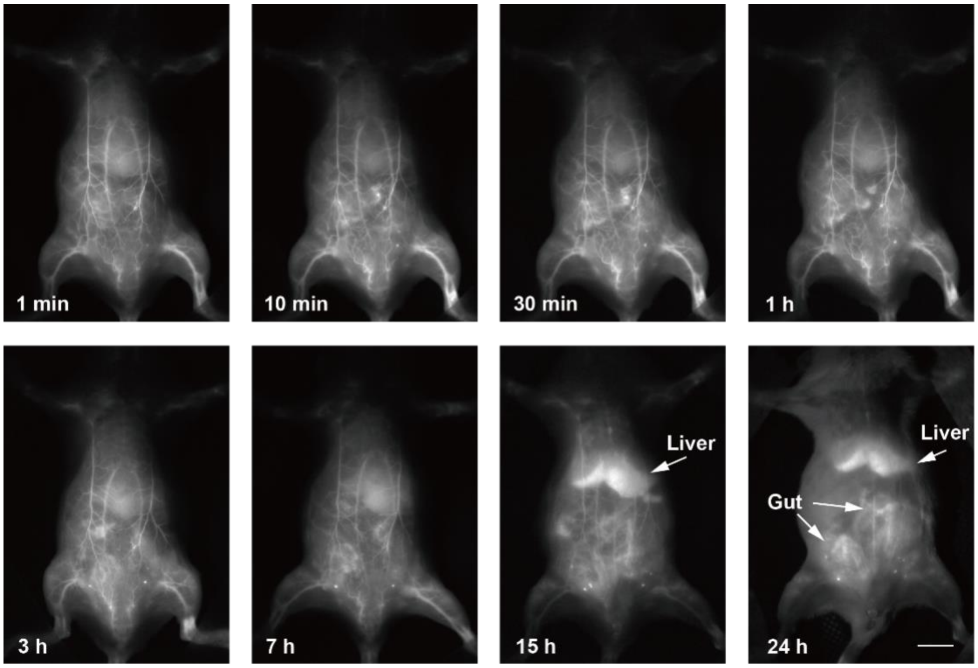

Supplementary Fig. 30. The whole-body imaging of mice after intravenous injection of 2FT-*o*CB dots within 24 hours. Scale bar, 10 mm.

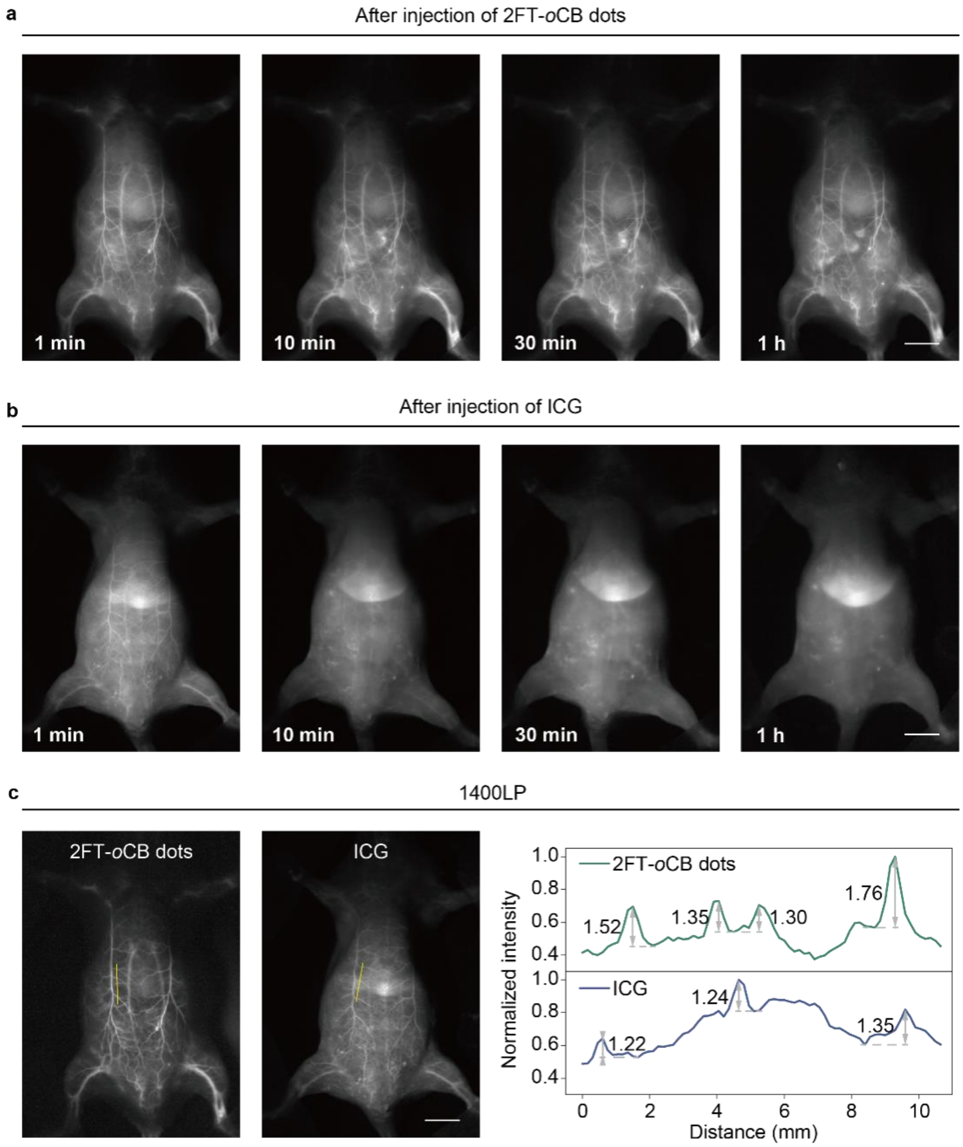

Supplementary Fig. 31. **The NIR-II fluorescence imaging comparison after intravenous injection of the 2FT-*o*CB dots and ICG.** (a) The images of mouse vessels using 2FT-*o*CB dots with 1300LP collection at various time points post-injection. (b) The images of mouse vessels using ICG with 1300LP collection at various time points post-injection. (c) The imaging comparison at 1 min post-injection in the NIR-IIx + NIR-IIb window. The numbers show the SBRs. Obviously, imaging using 2FT-*o*CB dots possesses better performance. Scale bars, 10 mm.

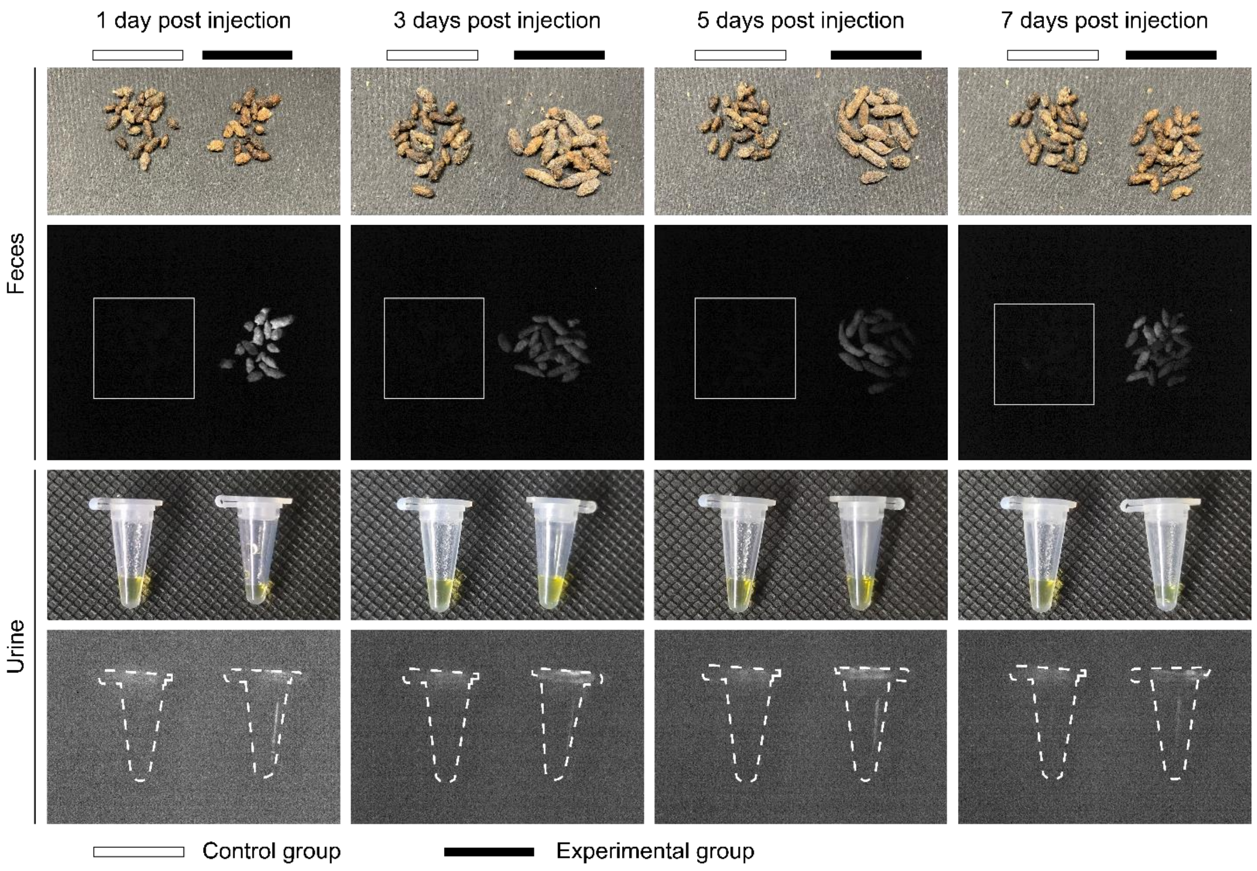

Supplementary Fig. 32. **The NIR-II fluorescence detection of mice feces and urine in one week.** The feces and urine from the mice with no treatment were considered as the control group; the feces and urine from the mice after intravenous injection of 2FT-*o*CB dots were regarded as the experimental group. The upper images were taken by the phone and the below images were taken by the InGaAs camera under the excitation of the 793 nm CW laser.

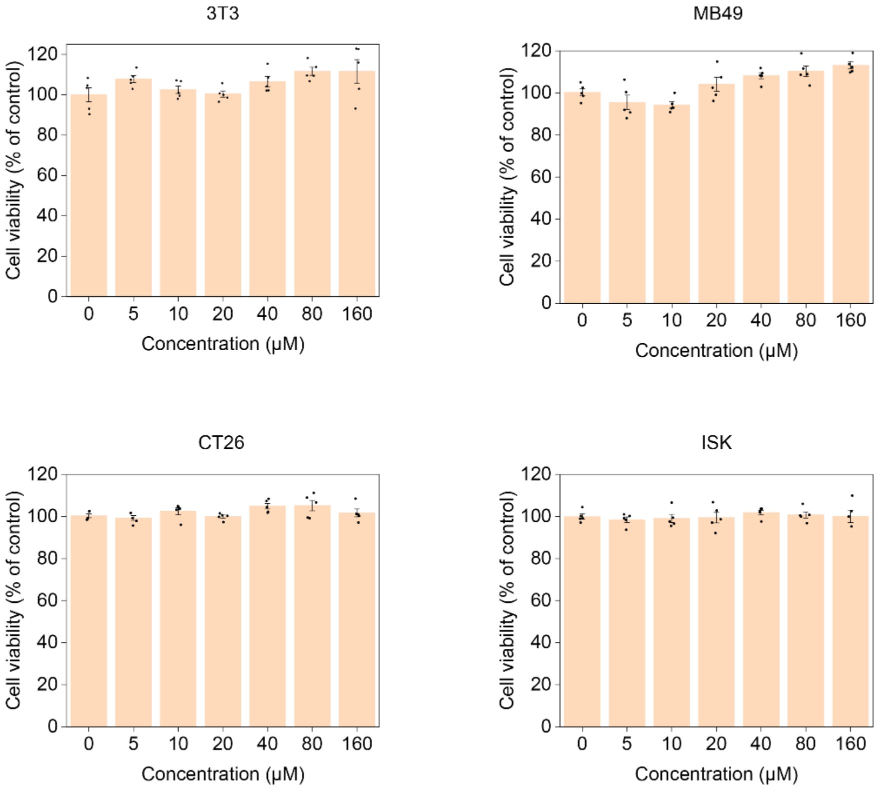

Supplementary Fig. 33. **The cell viability test of selected cell lines.** The 3T3, MB49, CT26, and ISK cells were studied here in 2FT-*o*CB dots solution of different concentrations. Mean ± SEM, n = 5.

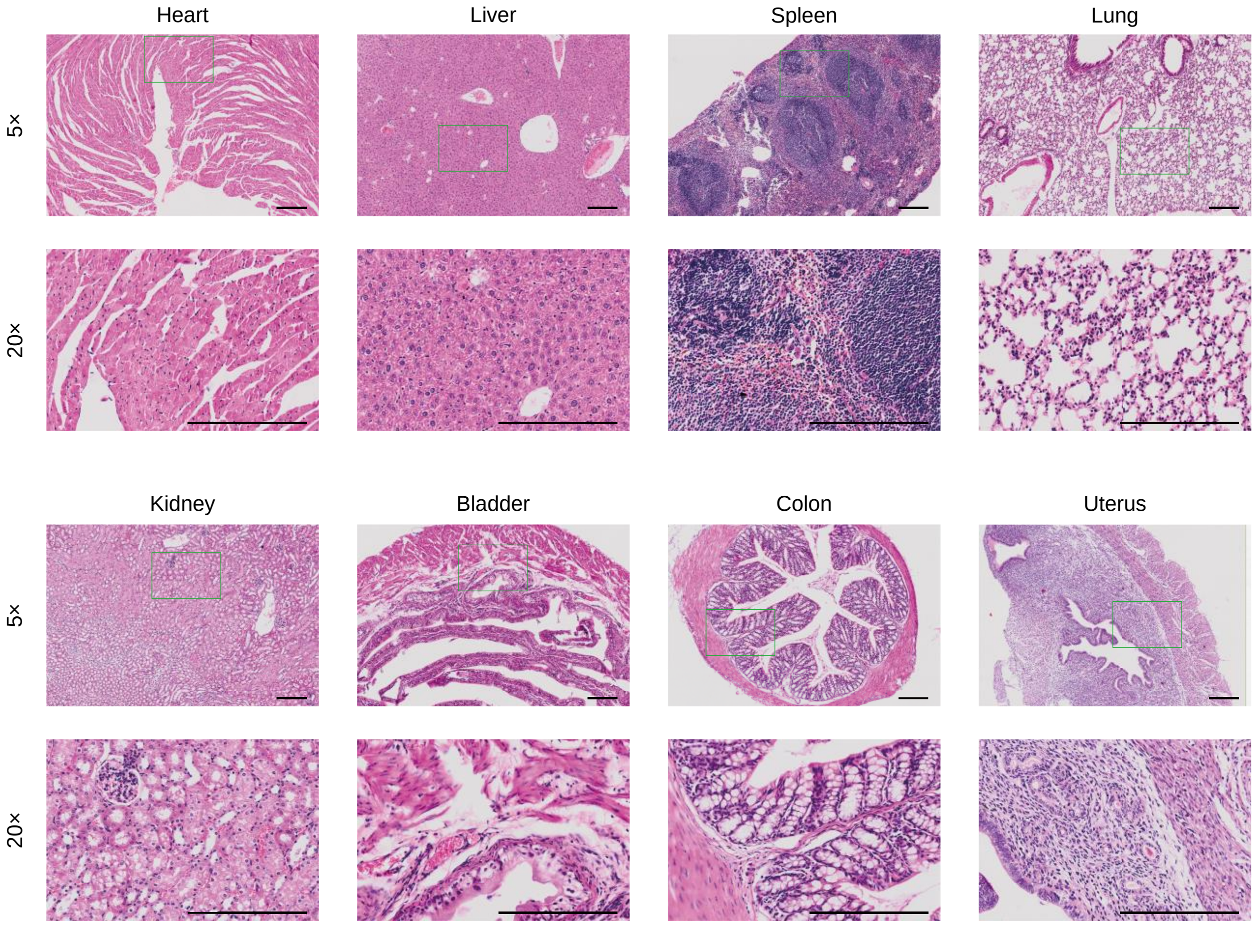

Supplementary Fig. 34. **Representative H&E staining images from control group.** The 20× images, selected by green boxes in the 5× images, are presented below the 5× images. n = 3 for the control group. Scale bar, 200 μm.

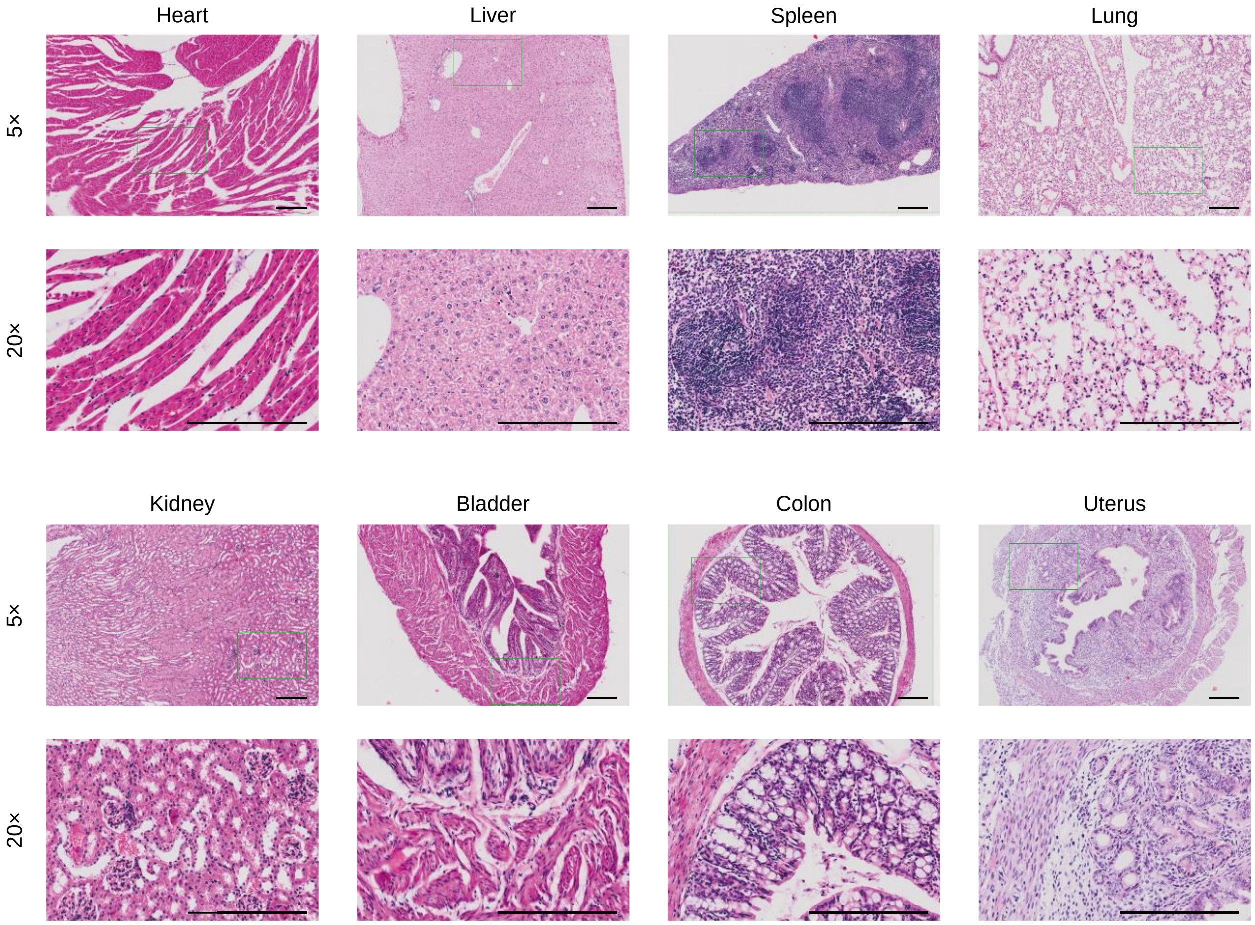

Supplementary Fig. 35. **Representative H&E staining images from experimental group.** The 20× images, selected by green boxes in the 5× images, are presented below the 5× images. n = 3 for the experimental group. Scale bar, 200 μm.

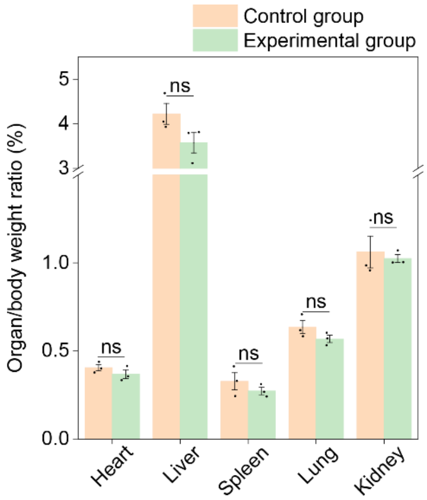

Supplementary Fig. 36. **The organ/body weight ratio of mice after 28 days of injection.** Group comparisons were made by two-tailed unpaired Student's T tests. Mean ± SEM, n = 3, ns $p > 0.05$. p

value = 0.2778 for the Heart; p value = 0.1195 for the Liver; p value = 0.3554 for the Spleen; p value = 0.1875 for the Lung; p value = 0.7169 for the Kidney.

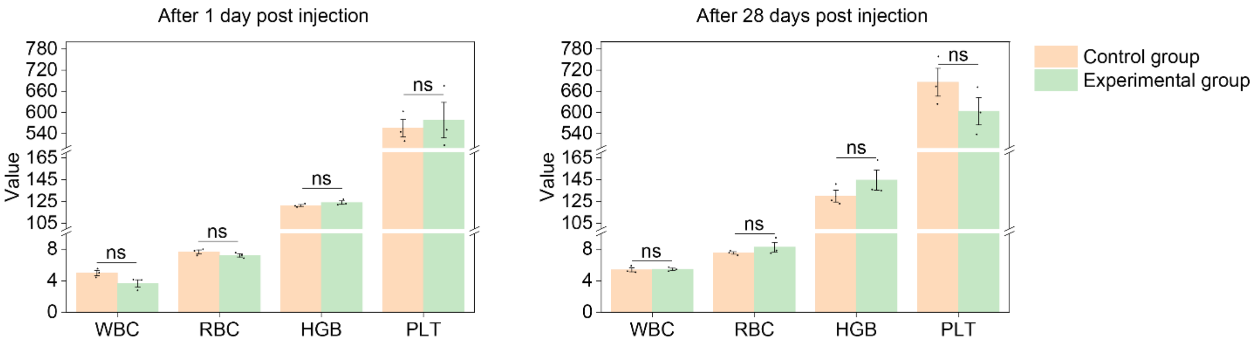

Supplementary Fig. 37. **The blood routine examination.** WBC, white blood cell (10^9/L); RBC, red blood cell (10^{12}/L); HGB, hemoglobin (g/L); PLT, platelet (10^9/L). Group comparisons were made by two-tailed unpaired Student's T tests. Mean ± SEM, n = 3, ns $p > 0.05$. p value = 0.0770 for the WBC after 1 day post injection; p value = 0.2268 for the RBC after 1 day post injection; p value = 0.2051 for the HGB after 1 day post injection; p value = 0.7122 for the PLT after 1 day post injection; p value = 0.8385 for the WBC after 28 days post injection; p value = 0.3082 for the RBC after 28 days post injection; p value = 0.2434 for the HGB after 28 days post injection; p value = 0.2104 for the PLT after 28 days post injection.

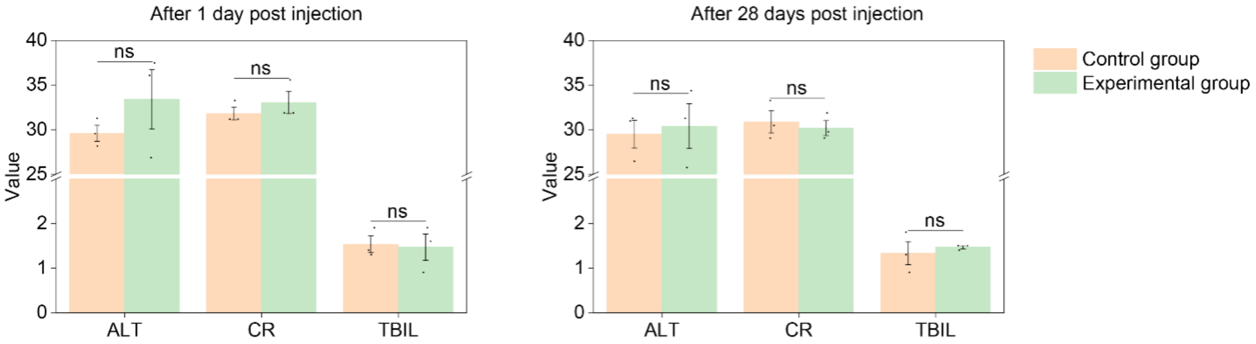

Supplementary Fig. 38. **The blood biochemistry test.** ALT, alanine transaminase (U/L); CR, creatinine (μM); TBIL, total bilirubin (μM). Group comparisons were made by two-tailed unpaired Student's T tests. Mean ± SEM, n = 3, ns $p > 0.05$. p value = 0.3317 for the ALT after 1 day post injection; p value = 0.4435 for the CR after 1 day post injection; p value = 0.8581 for the TBIL after 1 day post injection; p value = 0.7759 for the ALT after 28 days post injection; p value = 0.6638 for the CR after 28 days post injection; p value = 0.6382 for the TBIL after 28 days post injection.

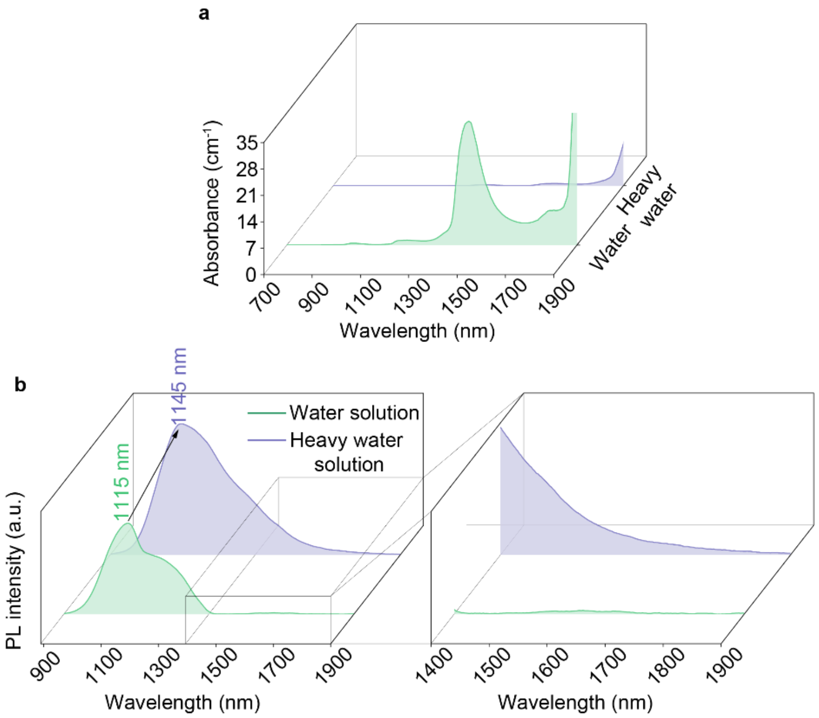

Supplementary Fig. 39. **The heavy water dispersion recovers the NIR-II emission of the fluorescent dots. (a)** The absorbance of water and heavy water in the NIR-I and NIR-II region. **(b)** The PL spectra of 2FT-*o*CB dots in water and heavy water with the same concentration and the enlarged spectra from 1400 nm to 1900 nm. a.u. here represents arbitrary units.

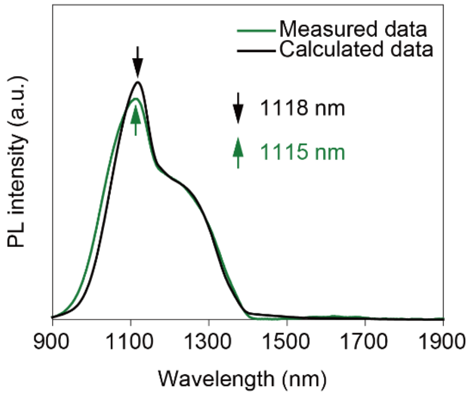

Supplementary Fig. 40. The comparison between the calculated spectrum and the measured spectrum of water solution. The calculated data was simulated *via* a fitted attenuation process with 95% confidence bounds using the emission property of 2FT-*o*CB dots in the heavy water and the absorption spectrum of water. Comparing the calculated data with the measured spectrum in water could directly reveal the leading role of solvent transference in the differences in emission spectra of 2FT-*o*CB dots in the two solvents. a.u. here represents arbitrary units.

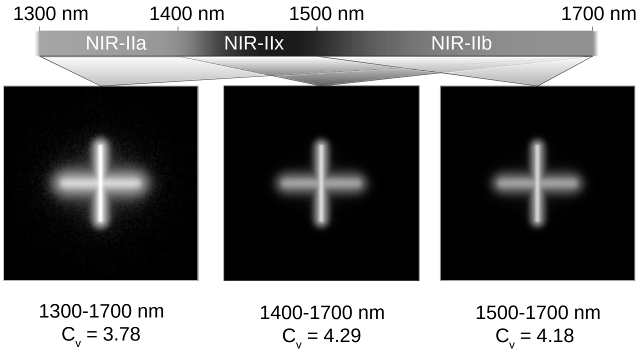

Supplementary Fig. 41. The imaging simulation of the 2FT-*o*CB dots (heavy water solution) in deep tissues via Monte Carlo method.

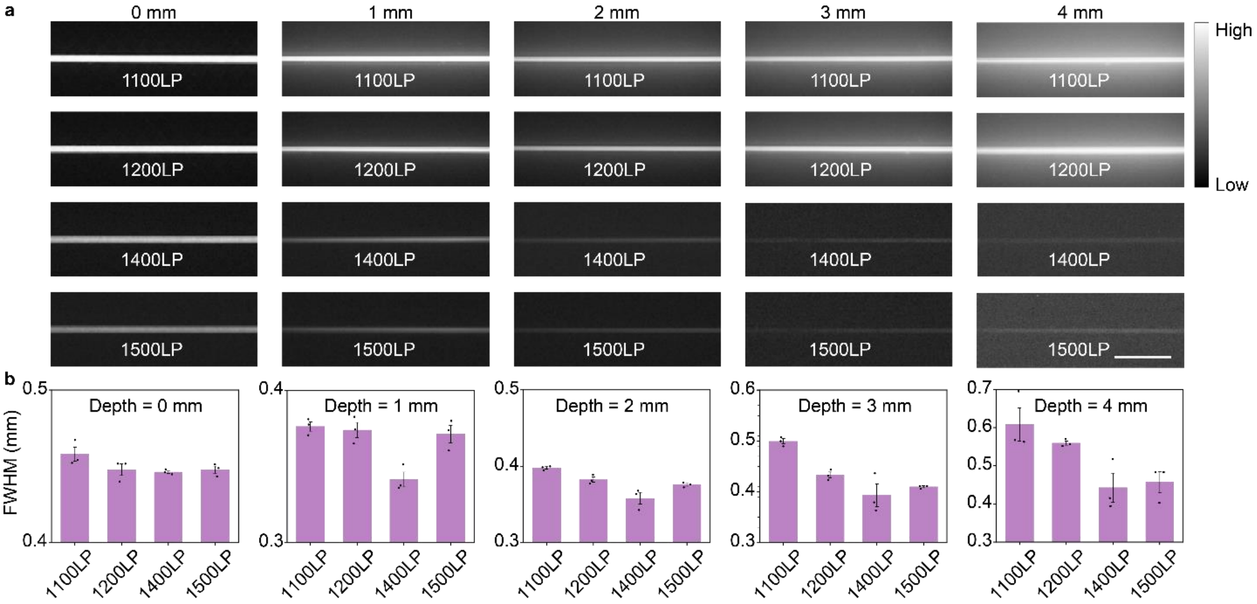

Supplementary Fig. 42. **The NIR-II fluorescence phantom imaging in different spectral regions. (a)** The phantom images of capillaries filled with the deuterium oxide dispersion of 2FT-*o*CB dots at depths of 0, 1, 2, 3, and 4 mm in 1% Intralipid® solution with 1100-, 1200-, 1400-, 1500-nm LP collection. Scale bar, 5 mm. **(b)** The FWHM measurements of the capillaries. Each sample was measured three times. Error bars indicate SEM (n = 3). Scale bar, 5 mm.

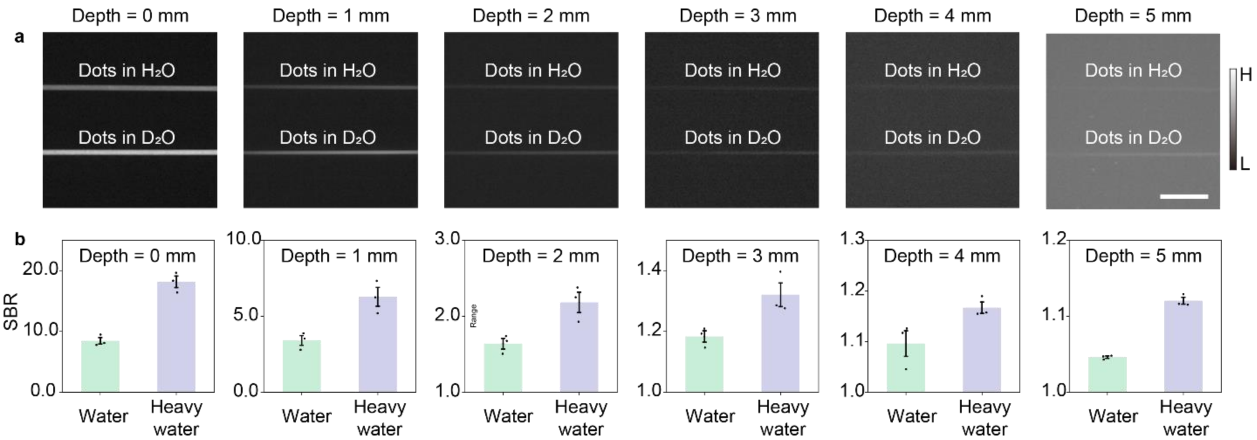

Supplementary Fig. 43. **The heavy water dispersion enhances the imaging SBR. (a)** The phantom images of capillaries filled with the hydrogen oxide (top) and deuterium oxide (bottom) dispersion of 2FT-*o*CB dots at depths of 0, 1, 2, 3, 4, and 5 mm in 1% Intralipid® solution with 1400-nm long-pass detection. Scale bar, 5 mm. **(b)** The SBR analyses of the capillaries. Each sample was measured three times. Error bars indicate SEM (n = 3).

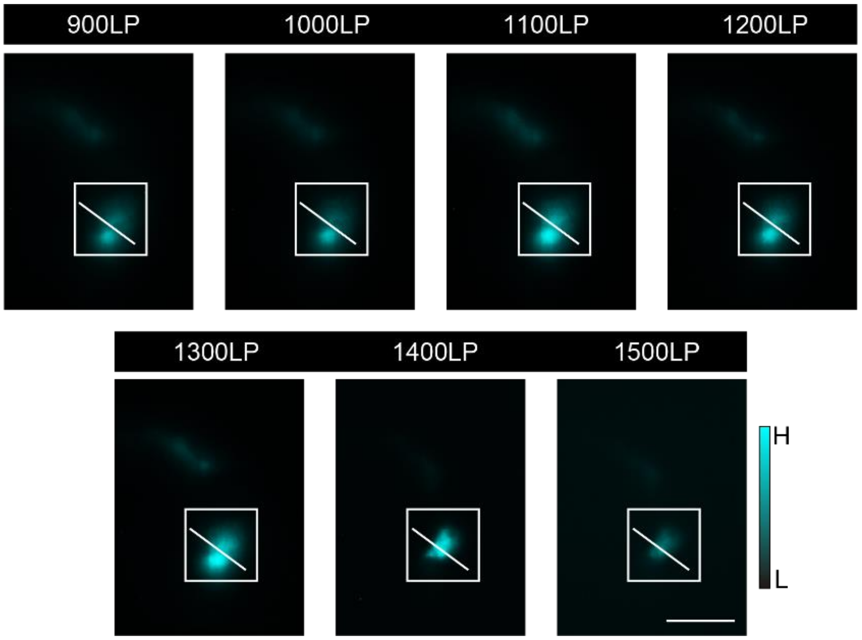

Supplementary Fig. 44. The images of fluorescence colonography (Scale bar, 5 mm) with colonic perfusion of 2FT-*o*CB dots in different NIR-II spectral regions.

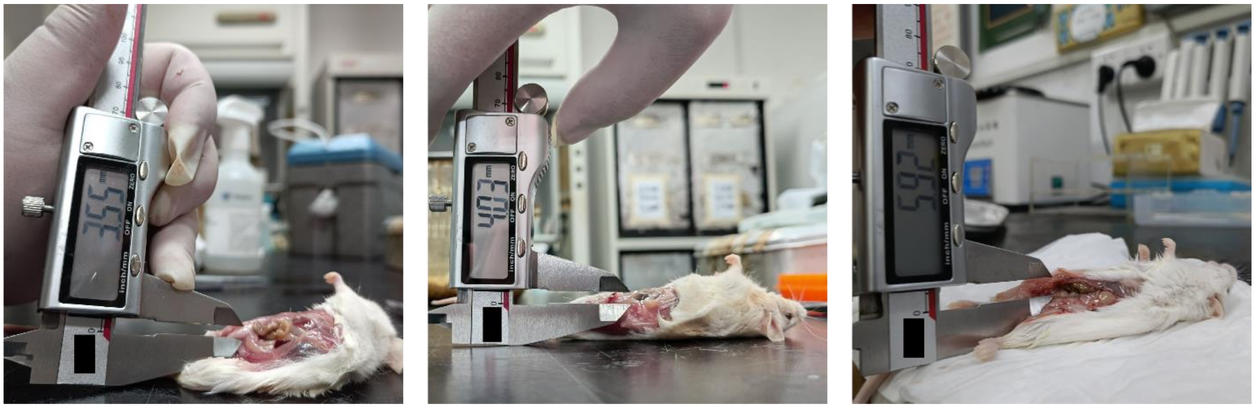

Supplementary Fig. 45. Tissue depth measurements of mouse colon. n = 3.

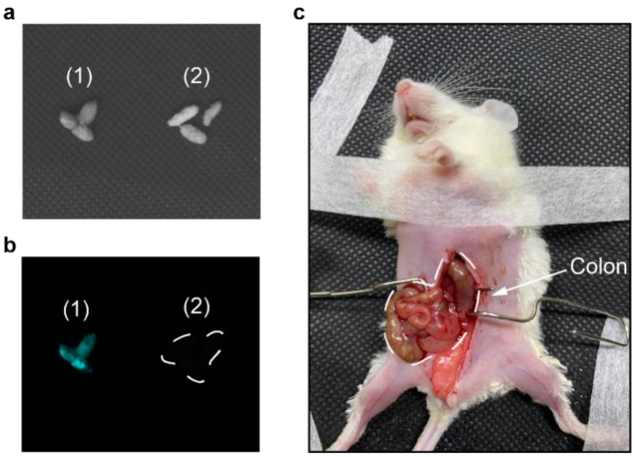

Supplementary Fig. 46. The **(a)** bright-field and **(b)** NIR-II fluorescence images of the feces from the mouse treated with (1) dots and (2) 1×PBS. **(c)** The picture of the mouse with the filling of the colon after opening the abdomen.

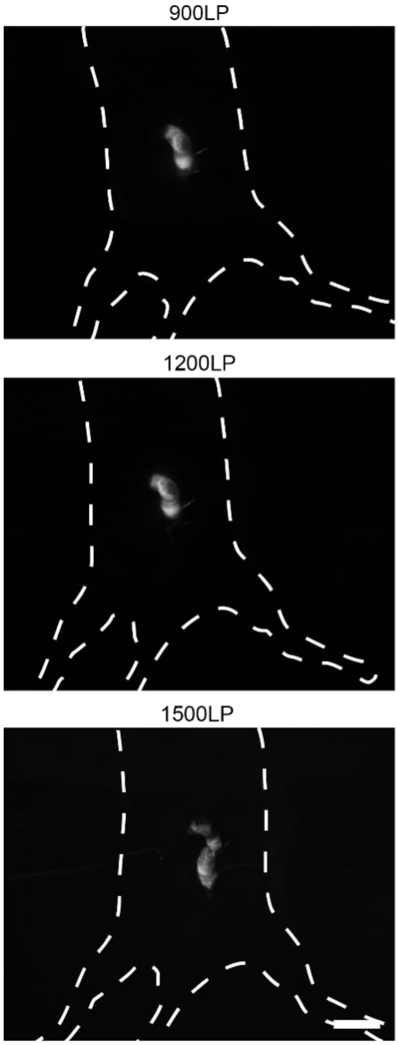

Supplementary Fig. 47. The NIR-II fluorescence colon images after opening the abdomen in different collection spectral regions. Scale bar, 5 mm.

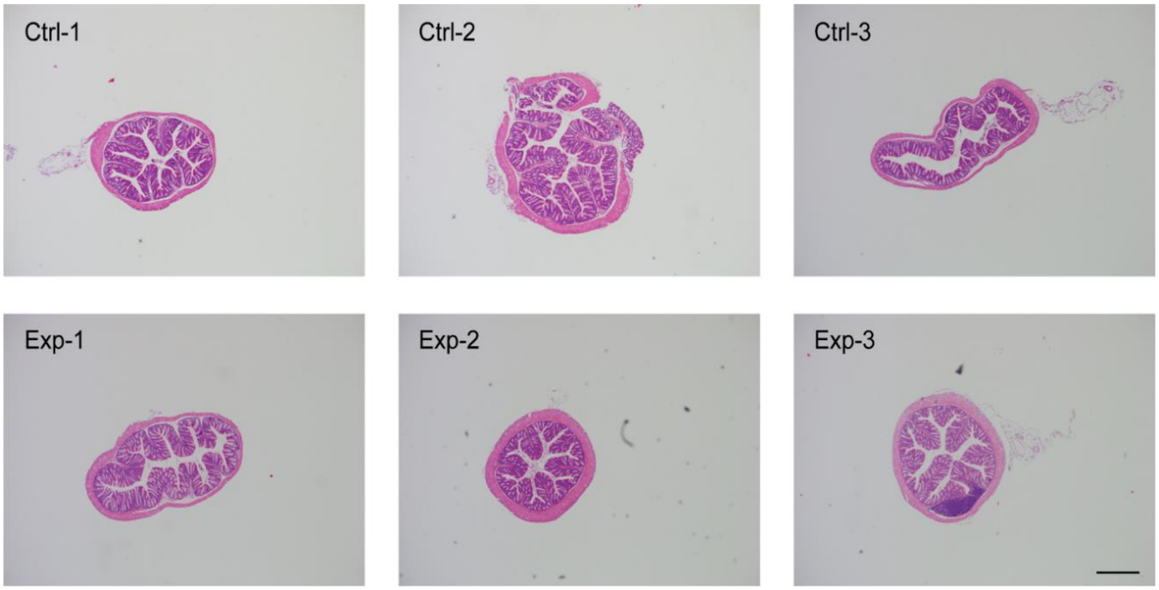

Supplementary Fig. 48. The H&E staining analysis of the colons from mice after colonic perfusion with 1×PBS (Control group: Ctrl-1, Ctrl-2 and Ctrl-3; n = 3) and 2FT-*o*CB dots (Experimental group: Exp-1, Exp-2 and Exp-3; n = 3). Scale bar, 500 μm.

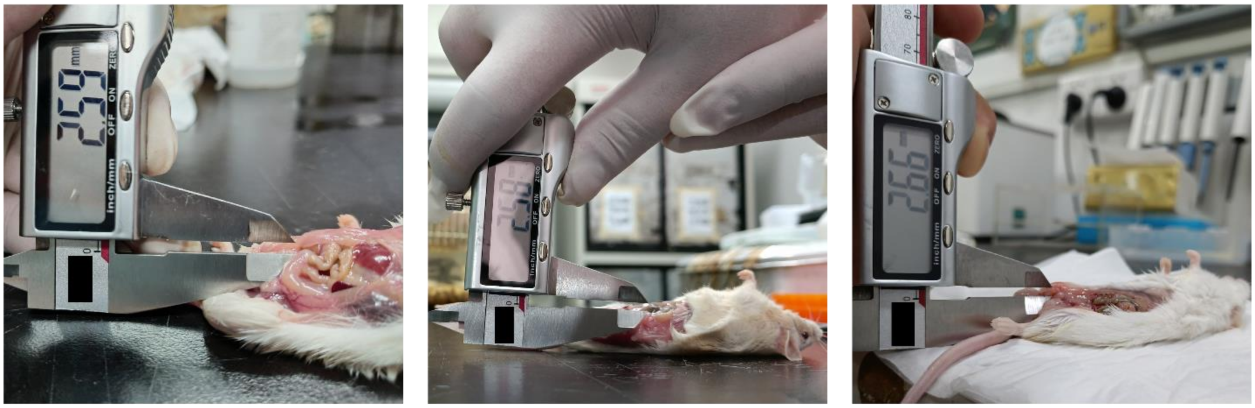

Supplementary Fig. 49. Tissue depth measurements of mouse bladder. n = 3.

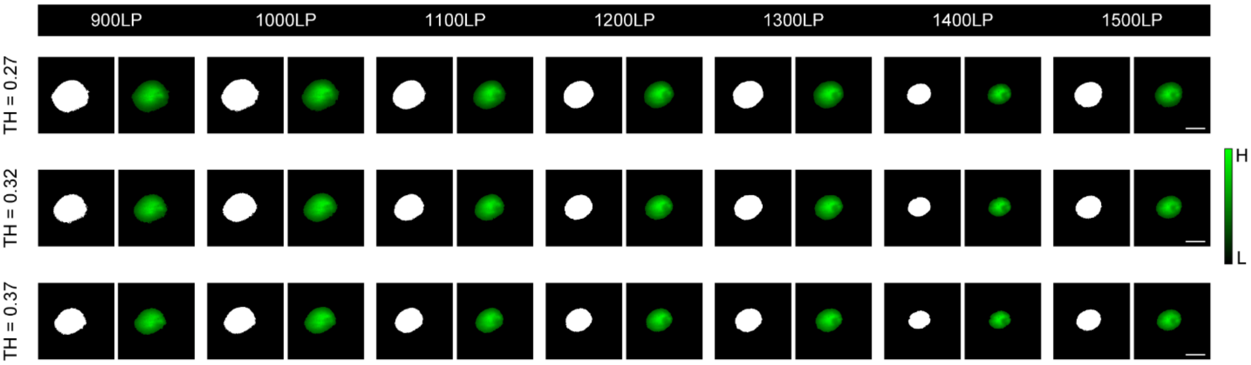

Supplementary Fig. 50. The binary and segmented bladder images in different NIR-II spectral regions with the TH (threshold value) of 0.27, 0.32 and 0.37. Scale bar, 5 mm.

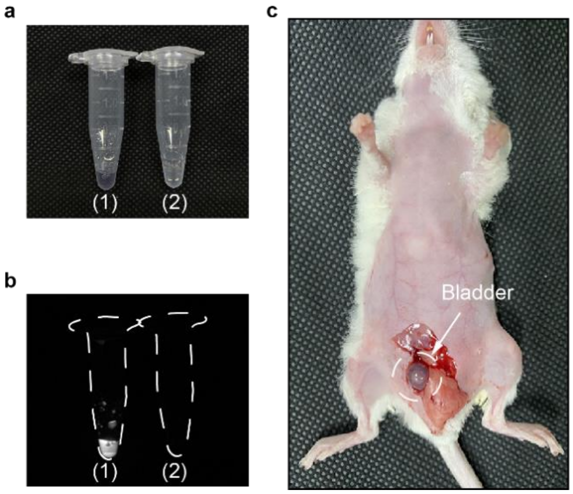

Supplementary Fig. 51. The **(a)** bright-field and **(b)** NIR-II fluorescence images of the two tubes containing (1) urine and (2) 1×PBS, respectively. **(c)** The picture of the mouse with the filling of the bladder after opening the abdomen.

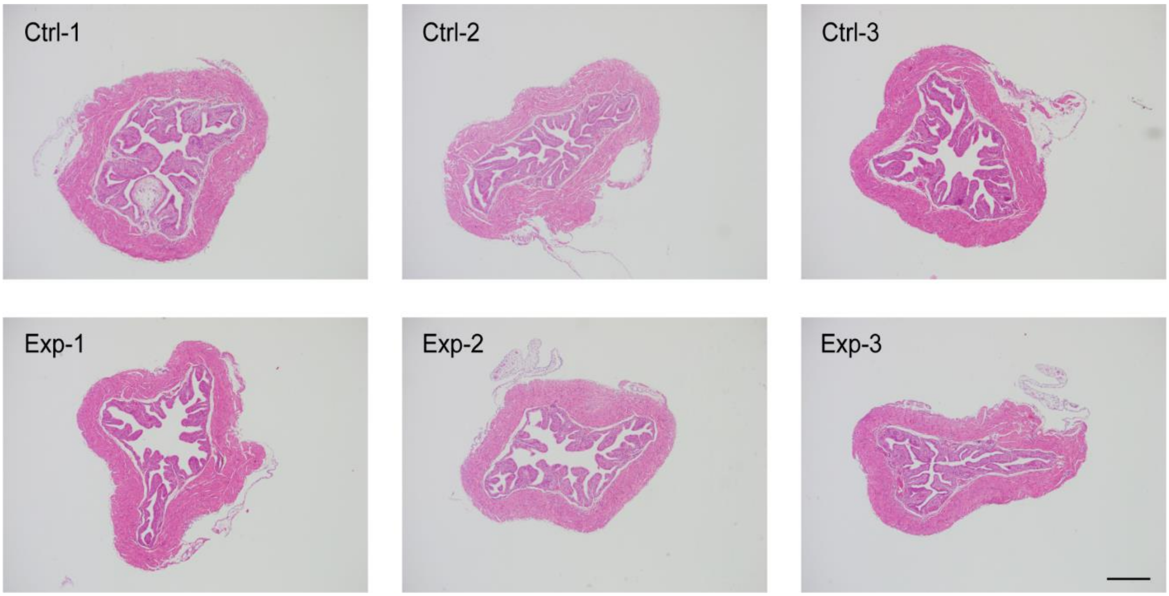

Supplementary Fig. 52. The H&E staining analysis of the bladders from mice after vesical perfusion with 1×PBS (Control group: Ctrl-1, Ctrl-2 and Ctrl-3; n = 3) and 2FT-*o*CB dots (Experimental group: Exp-1, Exp-2 and Exp-3; n = 3). Scale bar, 500 μm.

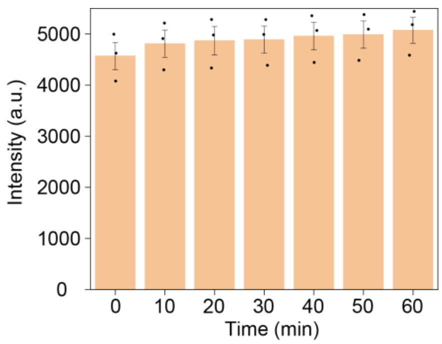

Supplementary Fig. 53. The stability test of the 2FT-*o*CB dots in mouse urine. Each sample was measured three times. Error bars indicate SEM (n = 3). a.u. here represents arbitrary units.

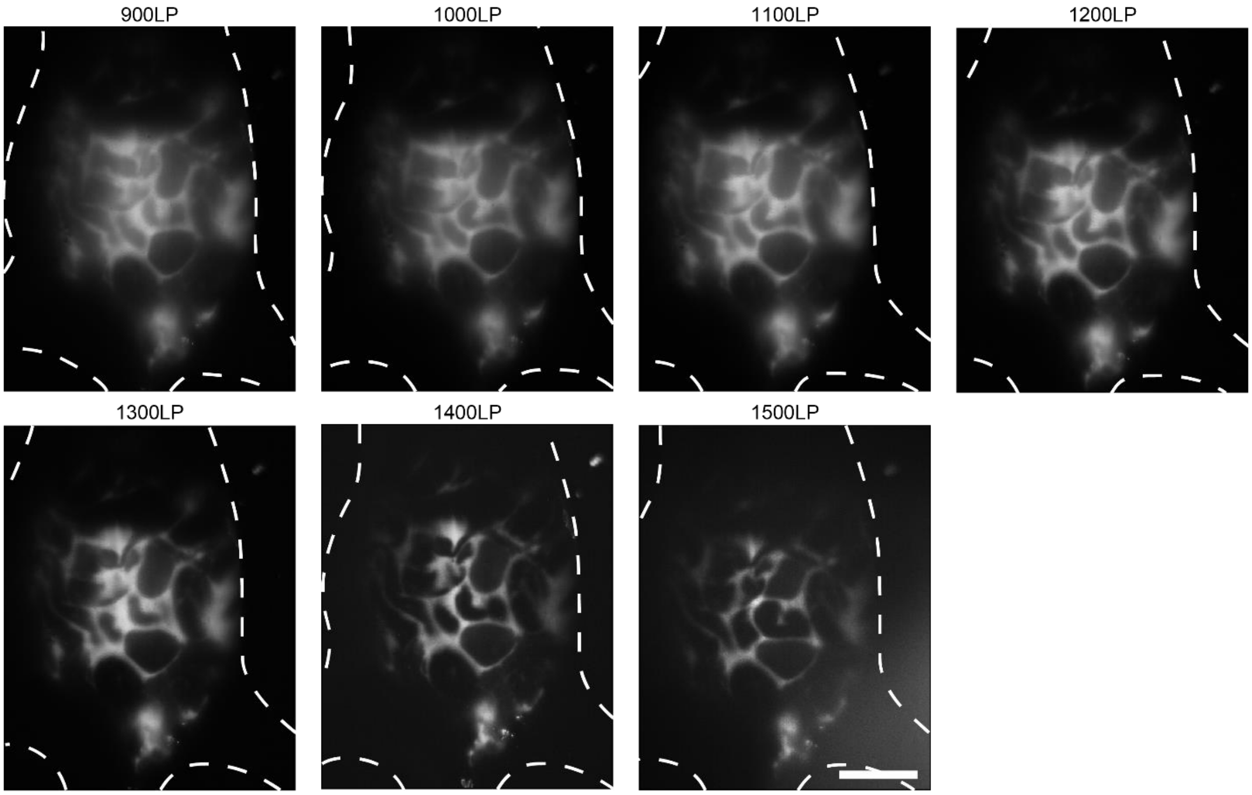

Supplementary Fig. 54. The whole-body imaging of a mouse with intraoperative bladder injury in different collection spectral regions. Scale bar, 10 mm.

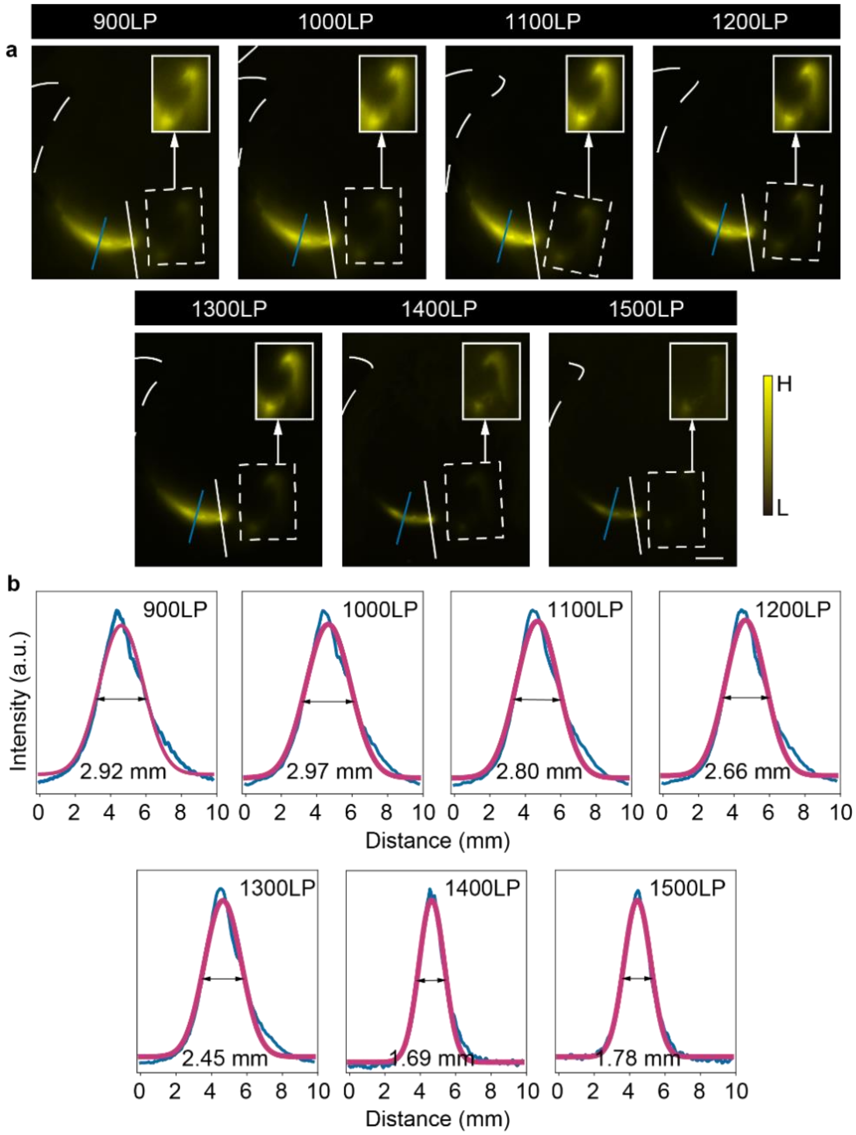

Supplementary Fig. 55. **(a)** The images of fluorescence hysterography with uterine perfusion of 2FT-*o*CB dots. Scale bar, 5 mm. The inserts are enhanced right side. Scale bar, 3 mm. **(b)** Cross-sectional fluorescence intensity profiles along the blue lines in (a). The white dashed lines in (a) describe the outline of the mouse body. a.u. here represents arbitrary units.

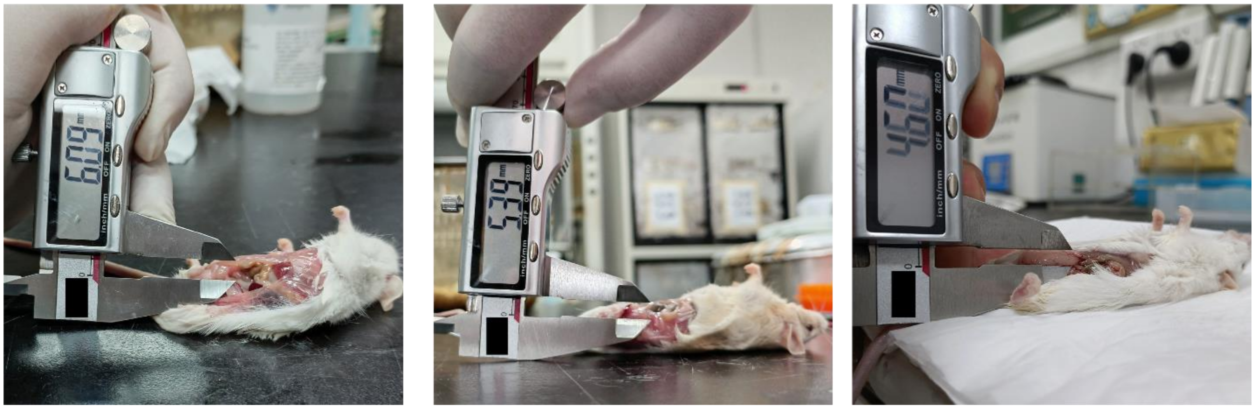

Supplementary Fig. 56. Tissue depth measurements of mouse uteruses. n = 3.

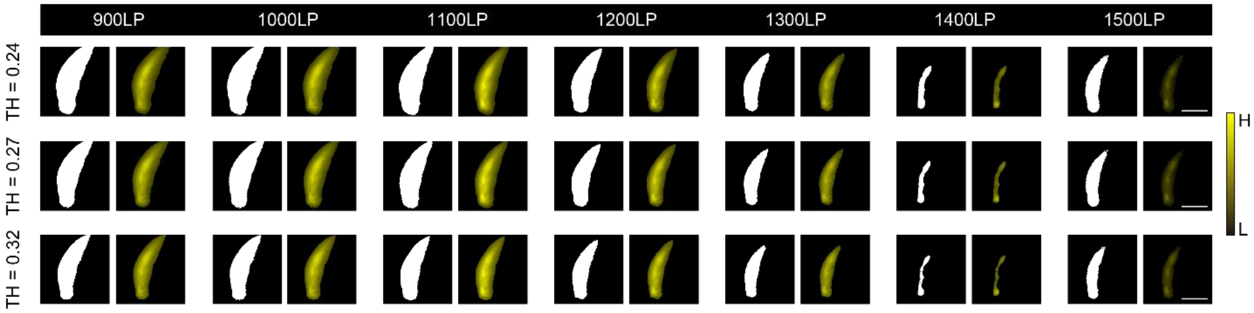

Supplementary Fig. 57. The binary and segmented uterus images with the TH of 0.27, 0.32 and 0.37. Scale bar, 5 mm.

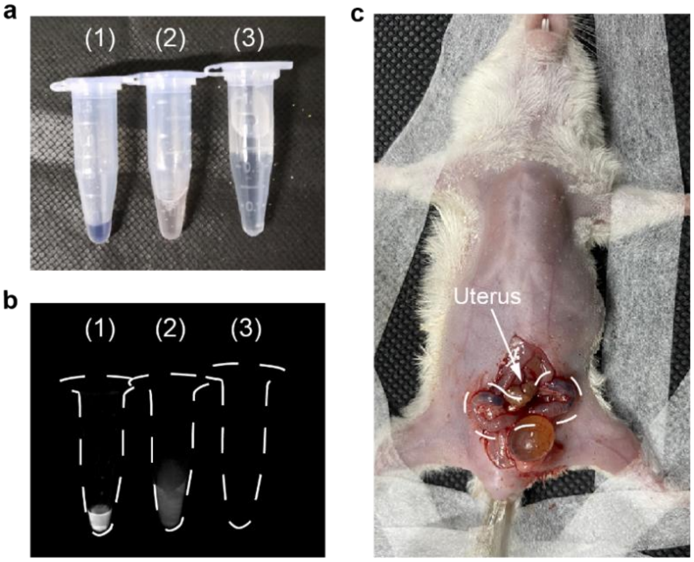

Supplementary Fig. 58. The **(a)** bright-field and **(b)** NIR-II fluorescence images of the three tubes containing the (1) liquid flowing out naturally from the uteruses, (2) intrauterine lavage fluid, and (3) 1×PBS, respectively. **(c)** The picture of the mouse with the filling of the uterus after opening the abdomen.

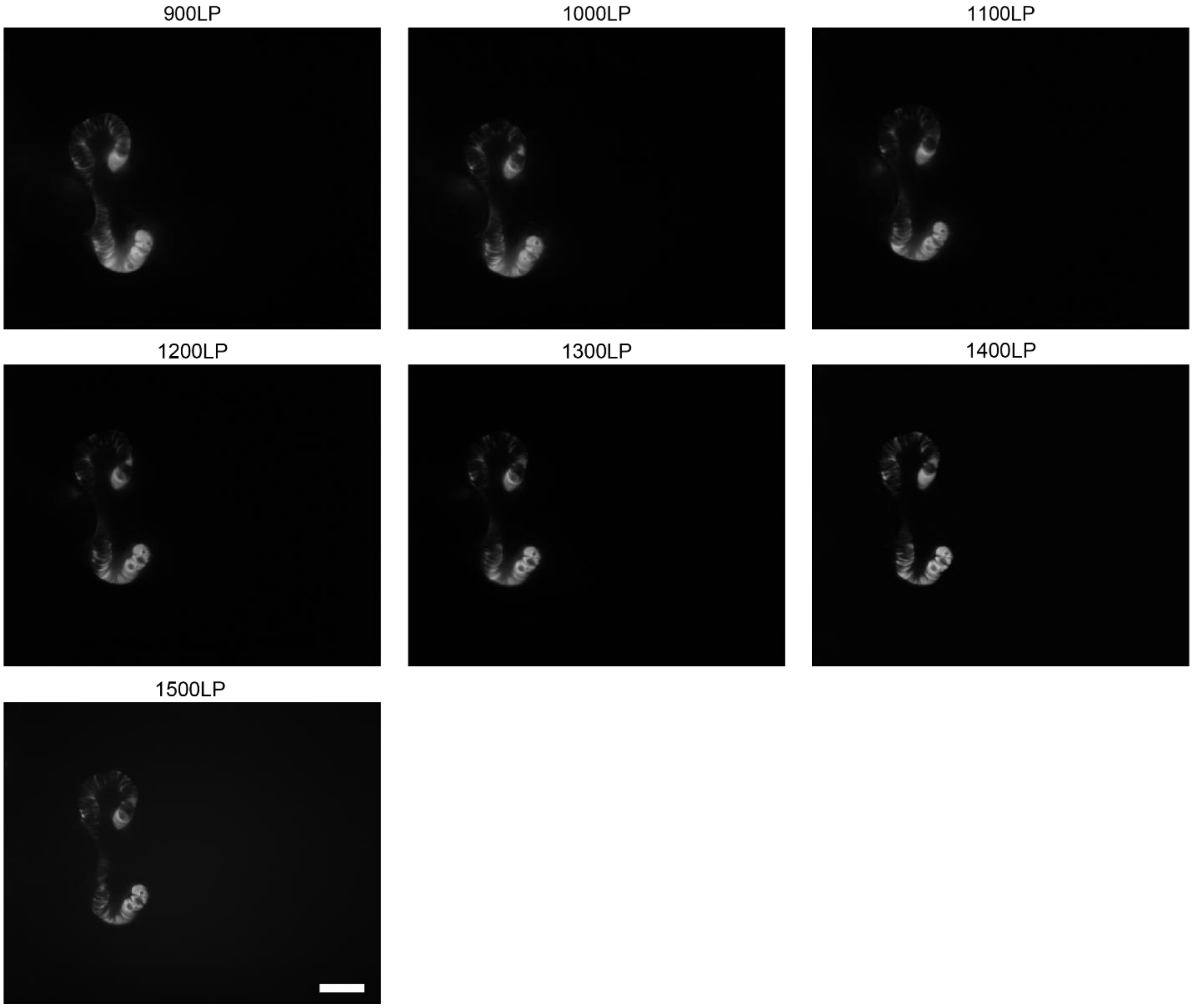

Supplementary Fig. 59. The NIR-II fluorescence uteruses images after opening the abdomen in different collection spectral regions. Scale bar, 5 mm.

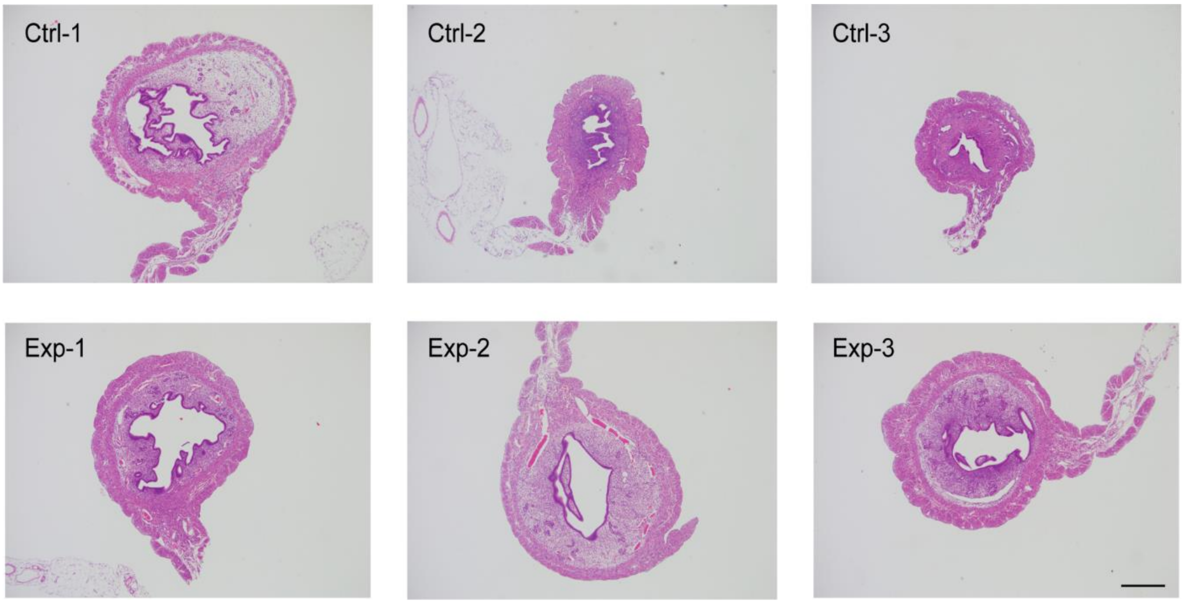

Supplementary Fig. 60. The H&E staining analysis of the uteruses from mice after uterine perfusion with 1×PBS (Control group: Ctrl-1, Ctrl-2 and Ctrl-3; n = 3) and 2FT-*o*CB dots (Experimental group: Exp-1, Exp-2 and Exp-3; n = 3). Scale bar, 500 μm.

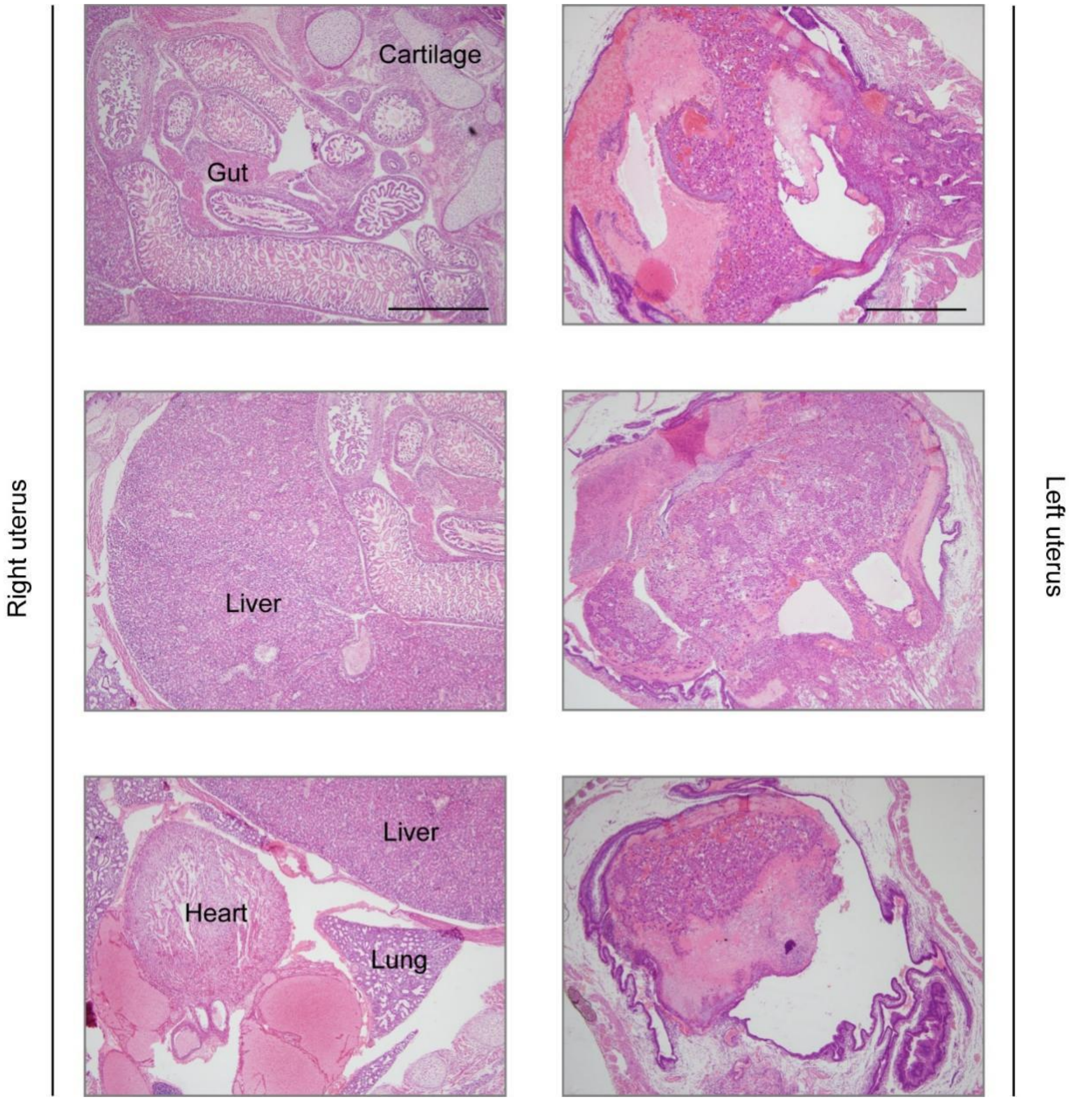

Supplementary Fig. 61. The H&E staining analysis of the fetus in the right uterus and the residual pregnancy tissue in the left uterus from mice. Scale bars, 500 μm. The results are representative of three independent experiments.

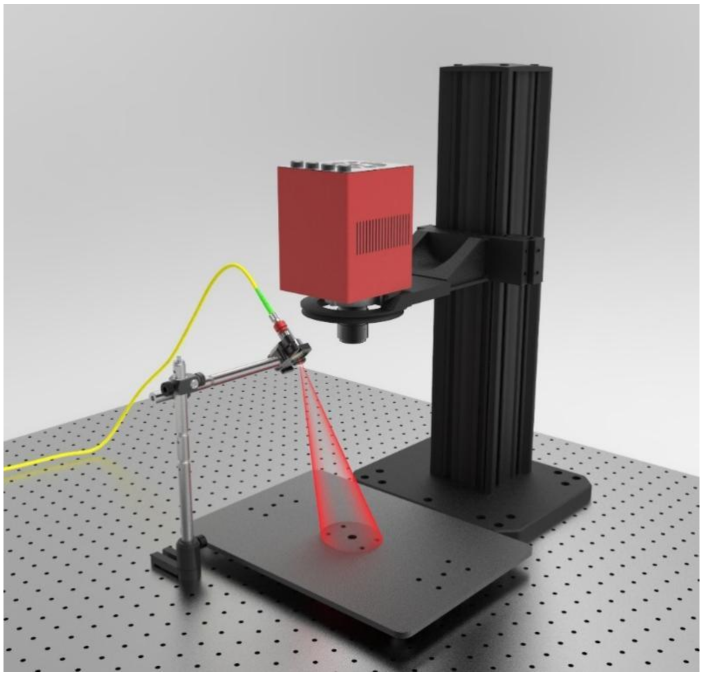

Supplementary Fig. 62. NIR-II fluorescence macro imaging system. To create this figure, we utilized Rhino 6 software to model the optical system and then rendered it using Keyshot 9.

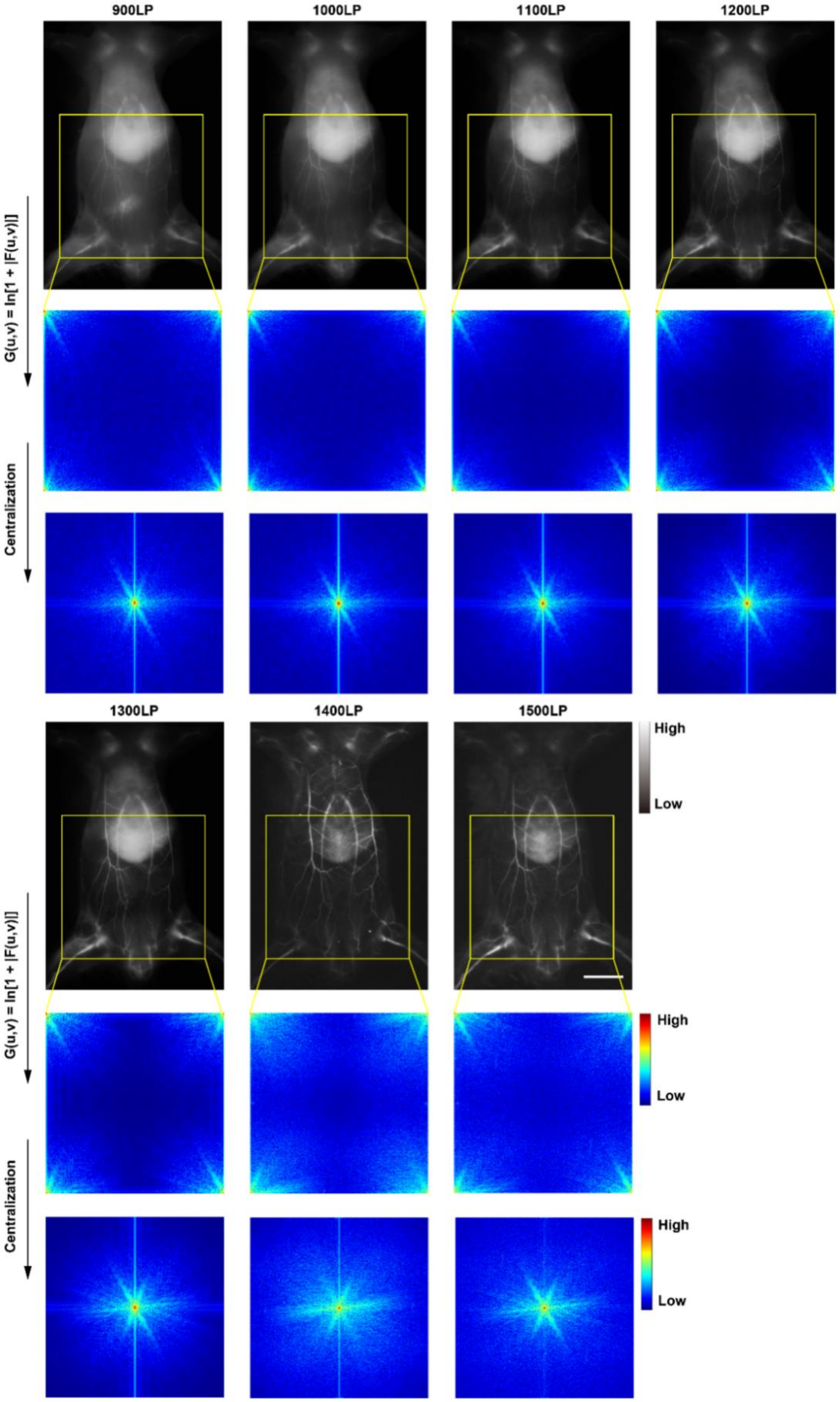

Supplementary Fig. 63. The whole image processing of the FFT results. Scale bar, 10 mm.

Supplementary Tables

Supplementary Table 1. Crystal data and structure refinement for 2FT-*o*CB.

**Empirical formula**	$C_{86}H_{80}N_6S_4$
**Formula weight**	1325.80
**Temperature/K**	170.0
**Crystal system**	monoclinic
**Space group**	C2/c
**a/Å**	36.1288(14)
**b/Å**	8.4689(4)
**c/Å**	26.0216(11)
**α/°**	90
**β/°**	102.928(2)
**γ/°**	90
**Volume/Å^3**	7760.0(6)
**Z**	4
**ρ_{calc}g/cm^3**	1.135
**μ/mm^{-1}**	0.169
**F(000)**	2808.0
**Crystal size/mm^3**	0.16 × 0.03 × 0.02
**Radiation**	MoKα (λ = 0.71073)
**2Θ range for data collection/°**	5.008 to 54.372
**Index ranges**	$-46 \le h \le 46, -10 \le k \le 10, -33 \le l \le 33$
**Reflections collected**	65676
**Independent reflections**	8588 [R_{int} = 0.0897, R_{sigma} = 0.0462]

Data/restraints/parameters	8588/111/493
Goodness-of-fit on F^2	1.024
Final R indexes [I>=2σ (I)]	R_1 = 0.0640, wR_2 = 0.1744
Final R indexes [all data]	R_1 = 0.0915, wR_2 = 0.1976
Largest diff. peak/hole / e Å^{-3}	0.61/-1.07

Supplementary Table 2. The power densities and the exposure times in the experiments.

Long-pass detection (LP)	**Whole-body vessel imaging**		**Fluorescence cystography**		**Fluorescence hysterography**		**Fluorescence colonography**	
	Power density (mW/cm^2)	Exposure time (ms)	Power density (mW/cm^2)	Exposure time (ms)	Power density (mW/cm^2)	Exposure time (ms)	Power density (mW/cm^2)	Exposure time (ms)
900	~20	50	~10	50	~15	50	~20	50
1000	~20	50	~10	50	~15	50	~20	50
1100	~40	50	~10	80	~20	100	~40	50
1200	~40	200	~20	80	~40	100	~40	100
1300	~100	500	~60	100	~60	200	~80	200
1400	~120	800	~100	500	~120	500	~160	500
1500	~120	1000	~100	500	~120	500	~160	500

Supplementary Table 3. The optical properties comparison between our previously developed NIR-II dyes in recent two years and the **2FT-*o*CB** designed in this work.

General design strategies/ Year/ Reference number	Dyes	Peak absorption wavelength [nm]	Peak emission wavelength [nm]	Peak molar absorption coefficient [×10^4 M^{-1} cm^{-1}]	NIR-II quantum yield (IR-26 as the reference, 0.5%)	Fluorescence imaging window [nm]
Enhancing D–A strength/ 2020/ [1]	TI	~558 (NPs)	~828 (NPs)	——	——	——
	TSI	~590 (NPs)	~942 (NPs)	——	——	——
	TSSI	~590 (NPs)	~992 (NPs)	——	——	1020-1700

Enlarging D-A strength via increasing the thiophene numbers/ 2020/ [2]	TAM	581 (DMSO) 637 (Solid)	840 (DMSO) 818 (Aggregates) 850 (Solid)	——	0.2 (DMSO) 0.9 (Aggregates) 0.5 (Solid)	——
	TSAM	587 (DMSO) 641 (Solid)	933 (DMSO) 874 (Aggregates) 860 (Solid)	——	0.1 (DMSO) 0.6 (Aggregates) 0.3 (Solid)	——
	TSSAM	595 (DMSO) 643 (Solid)	1022 (DMSO) 950 (Aggregates) 884 (Solid)	——	0.1 (DMSO) 0.5 (Aggregates) 0.2 (Solid)	1000-1700
Enlarging D-A strength and extending π-conjugation into a propeller-shaped structure/ 2020/ [3], [4]	ITT	592 (Ethanol)	735 (Ethanol)	2.6	——	——
	BITT	595 (Ethanol)	741 (Ethanol)	3.9	——	
	ITB	624 (Ethanol)	759 (Ethanol)	3.1	——	
	BITB	630 (Ethanol)	771 (Ethanol)	4.3	——	
Twisting the molecular structure and adding the planar blocks/ 2020/ [5]	pNIR-1	870 (THF)	1112 (THF)	0.717	0%	——
	pNIR-2	700 (THF)	1010 (THF)	0.326	3.2%	——
	pNIR-3	663 (THF) 688 (NPs)	907 (THF) 925 (NPs)	1.06	1.9%	1000-1700/ 1319-1700
	pNIR-4	709 (THF) 750 (NPs)	1080 (THF) 1040 (NPs)	0.573	2.2%	1000-1700/ 1319-1700
Twisting the molecular structure via triphenylamine-based alkylthiophene motif/	2TT-*o*C6B	~692 (THF); 730 (NPs)	~1080 (THF); 1034 (NPs)	2.49	8.4% (0.09% beyond 1500 nm)	——
	2TT-*o*C26B	~692 (THF); 730 (NPs)	~1080 (THF);	2.25	11.5% (0.12%	1500-1700

2020/ [6], [7], [8]			1031 (NPs)		beyond 1500 nm)	
	2TT-*o*C610B	~700 (THF); 730 (NPs)	~1070 (THF); 1029 (NPs)	2.13	9.1% (0.11% beyond 1500 nm)	——
Adding the planar blocks/ 2020/ [9], [10]	TT1-*o*CB	700 (THF); 732 (NPs)	~992 (THF); 1002 (NPs)	1.90	8.6% (0.029% beyond 1500 nm)	——
	TT2-*o*CB	712 (THF); 752 (NPs)	~992 (THF); 1020 (NPs)	2.19	7.8% (0.033% beyond 1500 nm)	——
	TT3-*o*CB	722 (THF); 784 (NPs)	~993 (THF); 1062 (NPs)	2.36	4.6% (0.031% beyond 1500 nm)	1500-1700
Enlarging the conjugation length and distorting the backbone/ 2021/ [11]	TPA-DPTQ	623 (THF)	910 (THF)	1.16	——	——
	DPTA-DPTQ	900 (THF)	1125 (THF)	0.87	0.48% (THF) 0.005% (NPs)	——
	DPBTA-DPTQ	806 (THF); 817 (NPs)	1120 (THF); 1125 (NPs)	1.04	0.16% (THF) 0.45% (NPs)	1000-1700
Enlarging absorption reservoir/ 2021/ [12]	TADAT	710 (THF); 754 (NPs)	986 (THF); 1114 (NPs)	16 (at 808 nm, in THF); 23 (at 808 nm, in aggregates)	0.2%	1250-1700
	TDADT	630/760 (THF); 838 (NPs)	986 (THF); 1275 (NPs)	15 (at 808 nm, THF); 21 (at 808 nm, in aggregates)	0.1%	1250-1700
——/ 2021/ [13], [14]	OTPA-BBT	770 (THF); ~700 (NPs)	~1000 (NPs)	~5	13.6% (0.12% beyond 1500 nm)	1100-1700/ 1500-1700
Changing the shielding units or motors/ 2022/ [15]	Alkoxy-BT-DPTQ	872 (THF)	~1100 (THF)	1.10	0.11 %	——
	TPE-BT-DPTQ	830 (THF)	~1100 (THF)	1.15	0.15 %	——

	TPA-BT-DPTQ	850 (THF)<br>853 (NPs)	~1100 (THF)<br>1117 (NPs)	1.04	0.13 %	1000-1700
——/<br>2022/<br>[16]	TPE-BT-BBTD	975 (THF);<br>958 (NPs)	1264 (THF);<br>1297 (NPs)	1.22 (at 980 nm)	0.02%	1200-1700
Twisting the skeletons and replacing the S atom with the Se atom/<br>2022/<br>[17]	NIR-820	820 (Chloroform)	1108 (Chloroform)	——	——	——
	NIR-920	920 (Chloroform);<br>~921 (NPs)	1188 (Chloroform);<br>~1258 (NPs)	——	0.02%	1200-1700/<br>1500-1700
Twisting the molecular structure via branched alkyl thiophene motif and facilitating D–A strength via diphenylamine fragments/<br>2022/<br>[18]	CTBT	682 (Chloroform);<br>680 (NPs)	858 (Chloroform);<br>976 (NPs)	1.75	16.92%	——
	DCTBT	704 (Chloroform);<br>716 (NPs)	995 (Chloroform);<br>1008 (NPs)	1.65	4.37%	
Enlarging acceptor rotors/<br>2022/<br>[19]	TPEDCPy	458 (DMSO)	660 (DMSO)	3.2	2.9%	——
	TPEDCQu	519 (DMSO)	730 (DMSO)	2.6	2.6%	
	TPEDCAc	570 (DMSO)	980 (DMSO)	1.8	0.4%	
Distorting of the backbone by introducing a rigid phenyl group grafted to the thiophene unit/<br>2022/<br>[20]	Ph	710 (THF);<br>730 (NPs)	1010 (THF);<br>1015 (NPs)	0.78	——	1550-1700
Reducing the D–A distance/<br>2022/<br>[21]	TBP-*b*-TPA	555 (THF)	688 (THF)	0.56	——	——
	TBP-*b*-DPA	663 (THF)	748 (THF)	0.12	3.0% (absolute QY in solid state);	——

					2.3% (absolute QY in solution state)	
	TBP-*b*-DFA	729 (THF); 740 (NPs)	888 (THF); 909 (NPs)	0.41	0.4% (absolute QY in solid state); 0.2% (absolute QY in solution state)	1000-1700
**Reducing the D–A distance and adjusting the donor/ This work**	**2FT-*o*CB**	**828** (THF); **846** (NPs)	**1215** (THF); **1135** (NPs in heave water)	**2.3**	0.95% beyond 900 nm, **but 0.11% beyond 1400 nm**	**1400-1700/ 1500-1700**

Supplementary Table 4. The optical properties comparison between the current AIE dyes for bioimaging beyond 1400/1500 nm (including but not limited to our contributions) and the **2FT-*o*CB** designed in this work.

Dyes/ Year/Reference number	Peak absorption wavelength [nm]	Peak emission wavelength [nm]	Peak molar absorption coefficient [$\times 10^4$ M^{-1} cm^{-1}]	NIR-II QY (IR-26 as the reference, 0.5 %)	Fluorescence imaging window [nm]
2TT-*o*C26B/ 2020/ [6], [7], [8]	~692 (THF) 730 (NPs)	~1080 (THF); 1031 (NPs)	2.25	0.12% beyond 1500 nm	1500-1700
TT3-*o*CB/ 2020/ [9], [10]	722 (THF); 784 (NPs)	~993 (THF); 1062 (NPs)	2.36	0.031% beyond 1500 nm	1500-1700
HQL2/ 2020/ [22]	~710 (DCM) ~720 (NPs)	1044 (DCM) ~993 (NPs)	1.98 (DCM); 5.44 (NPs)	0.002% beyond 1550 nm	1550-1700
HL3/ 2020/ [23]	~720 (THF); 750 (NPs)	1050 (THF); 1050 (NPs)	0.7 (THF); 0.93 (NPs)	0.05% beyond 1550 nm	1550-1700
OTPA-BBT/ 2021/ [13], [14]	770 (THF); ~700 (NPs)	~1000 (NPs)	~5	0.12% beyond 1500 nm	1500-1700

HY4/ 2022/ [24]	736 (THF); 748 (NPs)	1036 (THF); 1021 (NPs)	0.87	0.27% beyond 1500 nm	1500-1700
NIR-920/ 2022/ [17]	920 (Chloroform); ~921 (NPs)	1188 (Chloroform); ~1258 (NPs)	——	0.02% in the NIR-II window (**not beyond 1500 nm**)	1200-1700/ 1500-1700
Ph/ 2022/ [20]	710 (THF); 730 (NPs)	1010 (THF); 1015 (NPs)	0.78	——	1550-1700
**2FT-*o*CB/ This work**	**828 (THF); 846 (NPs)**	**1215 (THF); 1135 (NPs in heave water)**	**2.3**	**0.11% beyond 1400 nm**	**1400-1700/1500-1700**

Supplementary References

[1] Zhang, Z., *et al*. An all-round athlete on the track of phototheranostics: subtly regulating the balance between radiative and nonradiative decays for multimodal imaging-guided synergistic therapy. *Advanced Materials* **32**, 2003210 (2020).

[2] Xu, W., *et al*. Making the best use of excited-state energy: multimodality theranostic systems based on second near-infrared (NIR-II) aggregation-induced emission luminogens (AIEgens). *ACS Materials Letters* **2**, 1033–11040 (2020).

[3] Zhu, W., *et al*. Zwitterionic AIEgens: Rational Molecular Design for NIR-II Fluorescence Imaging-Guided Synergistic Phototherapy. *Advanced Functional Materials* **31**, 2007026 (2021).

[4] Ding, K., *et al*. Photo-enhanced chemotherapy performance in bladder cancer treatment via albumin coated AIE aggregates. *ACS Nano* **16**, 7535–7546 (2022).

[5] Liu, S., *et al*. Planar and twisted molecular structure leads to the high brightness of semiconducting polymer nanoparticles for NIR-IIa fluorescence imaging. *Journal of the American Chemical Society* **142**, 15146–115156 (2020).

[6] Li, Y., *et al*. Design of AIEgens for near-infrared IIb imaging through structural modulation at molecular and morphological levels. *Nature Communications* **11**, 1255 (2020).

[7] Liu, S., *et al*. Constitutional isomerization enables bright NIR-II AIEgen for brain-inflammation imaging. *Advanced Functional Materials* **30**, 1908125 (2020).

[8] Liu, S., *et al*. A two-in-one Janus NIR-II AIEgen with balanced absorption and emission for image-guided precision surgery. *Materials Today Bio* **10**, 100087 (2021).

[9] Liu, S., *et al*. Incorporation of Planar Blocks into Twisted Skeletons: Boosting Brightness of Fluorophores for Bioimaging beyond 1500 Nanometer. *ACS Nano* **14**, 14228–14239 (2020).

[10] Wu, D., *et al*. Organic Dots with Large π-Conjugated Planar for Cholangiography beyond 1500 nm in Rabbits: A Non-Radioactive Strategy. *ACS Nano* **15**, 5011–5022 (2021).

[11] Yan, D., *et al.* Donor/π-Bridge Manipulation for Constructing a Stable NIR-II Aggregation-Induced Emission Luminogen with Balanced Phototheranostic Performance. *Angewandte Chemie International Edition* **60**, 26769 (2021).

[12] Li, Y., *et al.* Enlarging the Reservoir: High Absorption Coefficient Dyes Enable Synergetic Near Infrared-II Fluorescence Imaging and Near Infrared-I Photothermal Therapy. *Advanced Functional Materials* **31**, 2102213 (2021).

[13] Feng, Z., *et al*. Biologically Excretable Aggregation-Induced Emission Dots for Visualizing Through the Marmosets Intravitally: Horizons in Future Clinical Nanomedicine. *Advanced Materials* **33**, 2008123 (2021).

[14] Yu, X., *et al*. Aggregation-induced emission dots assisted non-invasive fluorescence hysterography in near-infrared IIb window. *Nano Today* **39**, 101235 (2021).

[15] Yan, D., *et al.* Multimodal Imaging-Guided Photothermal Immunotherapy Based on a Versatile NIR-II Aggregation-Induced Emission Luminogen. *Angewandte Chemie International Edition* **61**, e202202614 (2022).

[16] Wang, M., *et al.* A versatile 980 nm absorbing aggregation-induced emission luminogen for NIR-II imaging-guided synergistic photo-immunotherapy against advanced pancreatic cancer. *Advanced Functional Materials* **32**, 2205371 (2022).

[17] Song, S., *et al.* Molecular engineering of AIE luminogens for NIR-II/IIb bioimaging and surgical navigation of lymph nodes. *Matter* **5**, 2847–2863 (2022).

[18] Li, D., *et al*. Synchronously boosting type-I photodynamic and photothermal efficacies via molecular manipulation for pancreatic cancer theranostics in the NIR-II window. *Biomaterials* **283**, 121476 (2022).

[19] Zhang, T., *et al*. Mitochondria-Targeting Phototheranostics by Aggregation-Induced NIR-II Emission Luminogens: Modulating Intramolecular Motion by Electron Acceptor Engineering for Multi-Modal Synergistic Therapy. *Advanced Functional Materials* **32**, 2110526 (2022).

[20] Wang, J., *et al*. Brain-Targeted Aggregation-Induced-Emission Nanoparticles with Near-Infrared Imaging at 1550 nm Boosts Orthotopic Glioblastoma Theranostics. *Advanced Materials* **34**, 2106082 (2022).

[21] Li, Y., *et al*. Molecular crystal engineering of organic chromophores for NIR-II fluorescence quantification of cerebrovascular function. *ACS Nano* **16**, 3323–3331 (2022).

[22] Li, Q., *et al*. Novel small-molecule fluorophores for in vivo NIR-IIa and NIR-IIb imaging. *Chemical Communications* **56**, 3289–3292 (2020).

[23] Li, Y., *et al*. Novel NIR-II organic fluorophores for bioimaging beyond 1550 nm. *Chemical Science* **11**, 2621–2626 (2020).

[24] Li, Y., *et al*. Small-molecule fluorophores for near-infrared IIb imaging and image-guided therapy of vascular diseases. *CCS Chemistry* **4**, 3735–3750 (2022).
