# Supplementary material for: Engineered NIR-II fluorophores with ultralong-distance molecular packing for high-contrast deep lesion identification
Source: Nat Commun. 2023 Aug 18;14:5017. doi: 10.1038/s41467-023-40728-6 (PMC10439134; doi:10.1038/s41467-023-40728-6)
Supplement: Supplementary file 3 — Reporting Summary [file 41467_2023_40728_MOESM3_ESM.pdf]

nature portfolio

Corresponding author(s): Jun Qian, Ben Zhong Tang

Last updated by author(s): Jun 16, 2023

Reporting Summary

Nature Portfolio wishes to improve the reproducibility of the work that we publish. This form provides structure for consistency and transparency in reporting. For further information on Nature Portfolio policies, see our Editorial Policies and the Editorial Policy Checklist.

Statistics

For all statistical analyses, confirm that the following items are present in the figure legend, table legend, main text, or Methods section.

n/a	Confirmed	
☐	☒	The exact sample size (n) for each experimental group/condition, given as a discrete number and unit of measurement
☐	☒	A statement on whether measurements were taken from distinct samples or whether the same sample was measured repeatedly
☐	☒	The statistical test(s) used AND whether they are one- or two-sided<br>*Only common tests should be described solely by name; describe more complex techniques in the Methods section.*
☒	☐	A description of all covariates tested
☒	☐	A description of any assumptions or corrections, such as tests of normality and adjustment for multiple comparisons
☐	☒	A full description of the statistical parameters including central tendency (e.g. means) or other basic estimates (e.g. regression coefficient) AND variation (e.g. standard deviation) or associated estimates of uncertainty (e.g. confidence intervals)
☐	☒	For null hypothesis testing, the test statistic (e.g. F, t, r) with confidence intervals, effect sizes, degrees of freedom and P value noted<br>*Give P values as exact values whenever suitable.*
☒	☐	For Bayesian analysis, information on the choice of priors and Markov chain Monte Carlo settings
☒	☐	For hierarchical and complex designs, identification of the appropriate level for tests and full reporting of outcomes
☒	☐	Estimates of effect sizes (e.g. Cohen's d, Pearson's r), indicating how they were calculated

Our web collection on statistics for biologists contains articles on many of the points above.

Software and code

Policy information about availability of computer code

Data collection	The fluorescence images were collected from the customized software of TEKWIN SYSTEM (5D640).
Data analysis	Quantitative analysis of the fluorescence images was performed using ImageJ software (Version 1.6.0, National Institutes of Health, USA). Data were analyzed using MATLAB R2020b, OriginPro 2018 (64-bit) and GraphPad Prism 8.0 (USA). The density functional theory calculations of the molecules were carried out using Gaussian 09 program.

For manuscripts utilizing custom algorithms or software that are central to the research but not yet described in published literature, software must be made available to editors and reviewers. We strongly encourage code deposition in a community repository (e.g. GitHub). See the Nature Portfolio guidelines for submitting code & software for further information.

Data

Policy information about availability of data

All manuscripts must include a data availability statement. This statement should provide the following information, where applicable:

- Accession codes, unique identifiers, or web links for publicly available datasets
- A description of any restrictions on data availability
- For clinical datasets or third party data, please ensure that the statement adheres to our policy

All data generated or analyzed during this study are included in this published article, Supplementary Information and Source Data file. The X-ray crystallographic data related to 2FT-oCB have been deposited at the Cambridge Crystallographic Data Centre (CCDC), under deposition number 2270673. These data can be

obtained free of charge from the CCDC via www.ccdc.cam.ac.uk/data_request/cif. There are no restrictions on data availability in the current work. Besides, Source data are provided with this paper.

Research involving human participants, their data, or biological material

Policy information about studies with human participants or human data. See also policy information about sex, gender (identity/presentation), and sexual orientation and race, ethnicity and racism.

Reporting on sex and gender	N/A
Reporting on race, ethnicity, or other socially relevant groupings	N/A
Population characteristics	N/A
Recruitment	N/A
Ethics oversight	N/A

Note that full information on the approval of the study protocol must also be provided in the manuscript.

Field-specific reporting

Please select the one below that is the best fit for your research. If you are not sure, read the appropriate sections before making your selection.

[x] Life sciences [ ] Behavioural & social sciences [ ] Ecological, evolutionary & environmental sciences

For a reference copy of the document with all sections, see nature.com/documents/nr-reporting-summary-flat.pdf

Life sciences study design

All studies must disclose on these points even when the disclosure is negative.

Sample size	Each group contained at least 3 mice. For cell data, we aimed to collect data from 5 biological replicates. The sample size was determined by the number of biological replicates necessary for ensuring statistical significance. Please see the figure legends for details.
Data exclusions	No data were excluded from the data analysis.
Replication	Experiments were repeated in at least three independent experiments with similar results. All experiments were reproduced to reliably support the conclusions stated in the manuscript.
Randomization	All samples were randomly allocated into experimental groups.
Blinding	The investigators were blinded to group allocation during data collection and analysis.

Reporting for specific materials, systems and methods

We require information from authors about some types of materials, experimental systems and methods used in many studies. Here, indicate whether each material, system or method listed is relevant to your study. If you are not sure if a list item applies to your research, read the appropriate section before selecting a response.

Materials & experimental systems

n/a	Involved in the study	
[x]	[ ]	Antibodies
[ ]	[x]	Eukaryotic cell lines
[x]	[ ]	Palaeontology and archaeology
[ ]	[x]	Animals and other organisms
[x]	[ ]	Clinical data
[x]	[ ]	Dual use research of concern
[x]	[ ]	Plants

Methods

n/a	Involved in the study	
[x]	[ ]	ChIP-seq
[x]	[ ]	Flow cytometry
[x]	[ ]	MRI-based neuroimaging

Eukaryotic cell lines

Policy information about cell lines and Sex and Gender in Research

Cell line source(s)	3T3 (CL-173) and CT26 (CRL-2638) cell lines were obtained from American Type Culture Collection (USA). MB49 (0s101398) cell line was purchased from ShangHai Zishi Biotech Co.,Ltd (China). ISK (TCH-C404) cell line was purchased from Haixing Biosciences (China)
Authentication	All cell lines were authenticated by Short Tandem Repeat test.
Mycoplasma contamination	Mycoplasma test was negative.
Commonly misidentified lines (See ICLAC register)	No commonly misidentified cell lines were used.

Animals and other research organisms

Policy information about studies involving animals; ARRIVE guidelines recommended for reporting animal research, and Sex and Gender in Research

Laboratory animals	The BALB/c nude mice (6-8 weeks old, ~20 g) and ICR mice (6-8 weeks old, ~20 g) involved in this work were purchased from the SLAC Laboratory Animal Co. Ltd. (Shanghai, China). All the experimental animals were housed under standard conditions in the Laboratory Animal Center of Zhejiang University, which were maintained and bred in specific pathogen-free conditions with a 12 h light and 12 h dark cycle, 25 °C room temperature and 50.0±5.0% humidity and had access to food and water ad libitum.
Wild animals	The study did not involve wild animals.
Reporting on sex	Female mice were used in the studies of fluorescence hysterography and intrauterine foreign body detection and there was no other sex bias in the animals used.
Field-collected samples	The study did not involve samples collected from the field.
Ethics oversight	All experimental procedures were approved by Animal Use and Care Committee at Zhejiang University. The BALB/c nude mice (~20 g) and ICR mice (~20 g) involved in this work were provided from the SLAC Laboratory Animal Co. Ltd. (Shanghai, China). All the experimental animals were housed under standard conditions in the Laboratory Animal Center of Zhejiang University, which were maintained and bred in specific pathogen-free conditions with a 12 h light and 12 h dark cycle, 25 °C room temperature and 50.0± 5.0% humidity and had access to food and water ad libitum. Female mice were used in the studies of fluorescence hysterography and intrauterine foreign body detection and there was no other sex bias in the animals used.

Note that full information on the approval of the study protocol must also be provided in the manuscript.
